# Functional and clinical relevance of VLA-4 (CD49d/CD29) in ibrutinib-treated chronic lymphocytic leukemia

**DOI:** 10.1084/jem.20171288

**Published:** 2018-02-05

**Authors:** Erika Tissino, Dania Benedetti, Sarah E.M. Herman, Elisa ten Hacken, Inhye E. Ahn, Kari G. Chaffee, Francesca Maria Rossi, Michele Dal Bo, Pietro Bulian, Riccardo Bomben, Elisabeth Bayer, Andrea Härzschel, Julia Christine Gutjahr, Massimiliano Postorino, Enrico Santinelli, Ayed Ayed, Francesco Zaja, Annalisa Chiarenza, Gabriele Pozzato, Alexandre Chigaev, Larry A. Sklar, Jan A. Burger, Alessandra Ferrajoli, Tait D. Shanafelt, Adrian Wiestner, Giovanni Del Poeta, Tanja Nicole Hartmann, Valter Gattei, Antonella Zucchetto

**Affiliations:** 1 Clinical and Experimental Onco-Hematology Unit, CRO Aviano National Cancer Institute, Aviano, Italy; 2 Hematology Branch, National Heart, Lung, and Blood Institute, National Institutes of Health, Bethesda, MD; 3 Department of Leukemia, The University of Texas MD Anderson Cancer Center, Houston, TX; 4 Mayo Clinic College of Medicine, Rochester, MN; 5 Third Medical Department with Hematology, Medical Oncology, Hemostaseology, Infectious Diseases, and Rheumatology, Oncologic Center, Paracelsus Medical University, Salzburg, Austria; 6 Cancer Cluster Salzburg, Salzburg Cancer Research Institute-Laboratory for Immunological and Molecular Cancer Research, Salzburg, Austria; 7 Division of Hematology, S. Eugenio Hospital and University of Tor Vergata, Rome, Italy; 8 Clinica Ematologica, Centro Trapianti e Terapie Cellulari “Carlo Melzi” DISM, Azienda Ospedaliera Universitaria S. Maria Misericordia, Udine, Italy; 9 Division of Hematology, Ferrarotto Hospital, Catania, Italy; 10 Department of Internal Medicine and Hematology, Maggiore General Hospital, University of Trieste, Trieste, Italy; 11 Department of Pathology and Cancer Center, University of New Mexico, Albuquerque, NM

## Abstract

The Bruton’s tyrosine kinase (BTK) inhibitor ibrutinib, which antagonizes B cell receptor (BCR) signals, demonstrates remarkable clinical activity in chronic lymphocytic leukemia (CLL). The lymphocytosis experienced by most patients under ibrutinib has previously been attributed to inhibition of BTK-dependent integrin and chemokine cues operating to retain the tumor cells in nodal compartments. Here, we show that the VLA-4 integrin, as expressed by CD49d-positive CLL, can be inside-out activated upon BCR triggering, thus reinforcing the adhesive capacities of CLL cells. In vitro and in vivo ibrutinib treatment, although reducing the constitutive VLA-4 activation and cell adhesion, can be overcome by exogenous BCR triggering in a BTK-independent manner involving PI3K. Clinically, in three independent ibrutinib-treated CLL cohorts, CD49d expression identifies cases with reduced lymphocytosis and inferior nodal response and behaves as independent predictor of shorter progression-free survival, suggesting the retention of CD49d-expressing CLL cells in tissue sites via activated VLA-4. Evaluation of CD49d expression should be incorporated in the characterization of CLL undergoing therapy with BCR inhibitors.

## Abstract (precis)

Tissino et al. demonstrate that in chronic lymphocytic leukemia, the VLA-4 (CD49d/CD29) integrin remains activable by B cell receptor stimulation also upon in vitro and in vivo ibrutinib exposure. Clinically, ibrutinib-treated CD49d-positive CLL patients experience reduced recirculation lymphocytosis and nodal response and inferior outcomes.

## Introduction

CD49d, the α chain of the CD49d/CD29 integrin heterodimer very late antigen 4 (VLA-4), expressed in ∼40% of chronic lymphocytic leukemia (CLL) cases, has emerged as one of the most relevant biological predictors of overall survival (OS) and progression-free survival (PFS) in CLL (Gattei et al., 2008; Shanafelt et al., 2008; Bulian et al., 2014). VLA-4 mediates both cell–cell and cell–matrix interactions in CLL-involved tissues by respectively binding to its ligands vascular cell adhesion molecule 1 (VCAM-1) and fibronectin (Ruoslahti, 1991; Hartmann et al., 2009).

VLA-4, usually present on the cell surface of resting leukocytes in an inactive conformation, can be activated in response to different stimuli, thus becoming competent for high-affinity and high-avidity interactions (Arana et al., 2008a). In particular, in normal B lymphocytes, stimuli originating from the B cell receptor (BCR) have been described to activate VLA-4 via inside-out signaling, an interplay occurring during the process of antigen-specific B cell differentiation that takes place in secondary lymphoid organs (Liu and Arpin, 1997). In this context, a proper BCR stimulation may trigger a cascade of molecular events eventually leading to increased VLA-4 activity and rescue of B cells from apoptosis through tonic interactions with VCAM-1–expressing follicular dendritic cells (Arana et al., 2008a).

Although not yet investigated in detail, such a BCR–VLA-4 interplay may be relevant in CLL particularly in the light of the central role played by the BCR pathway in this disease (Burger and Chiorazzi, 2013; Wiestner, 2015), as witnessed by the emerging remarkable clinical activities of several inhibitors that interfere with the action of key BCR-related intracellular enzymes (Woyach et al., 2012; Wiestner, 2014).

In particular, ibrutinib is an orally administered first-in-class covalent inhibitor of Bruton’s tyrosine kinase (BTK) that has been approved for treatment of CLL both in first-line and relapsed disease (Byrd et al., 2013; Farooqui et al., 2015). Clinically, ibrutinib yields a rapid shrinkage of tumor masses, a parallel redistribution of CLL cells from tissue sites into blood with a subsequent secondary lymphocytosis (Jones and Byrd, 2014). This pattern of clinical response, shared by all inhibitors targeting the BCR pathway, is thought to occur through inhibition of different integrin-dependent and/or chemokine-dependent microenvironmental interactions, including those mediated by VLA-4 (de Rooij et al., 2012; Ponader et al., 2012; Herman et al., 2015; Chen et al., 2016). Despite this, nothing has been reported regarding the modulation of VLA-4 activation by ibrutinib and the influence of VLA-4 expression/activation on ibrutinib response in vivo.

In the present study, by taking advantage of three different cohorts of in vivo ibrutinib-treated CLL and of a series of ibrutinib-naive primary CLL samples, we demonstrate that (1) the VLA-4 integrin can also be activated upon BCR triggering in ibrutinib-exposed CLL cells, (2) CLL cases expressing CD49d usually fail to display the canonical ibrutinib-induced lymphocytosis and experience a lower nodal response, and (3) CD49d expression is consistently associated with shorter PFS in the context of ibrutinib-treated CLL.

## Results

### BCR stimulation induces inside-out VLA-4 activation in CLL cells

**Figure 1.**
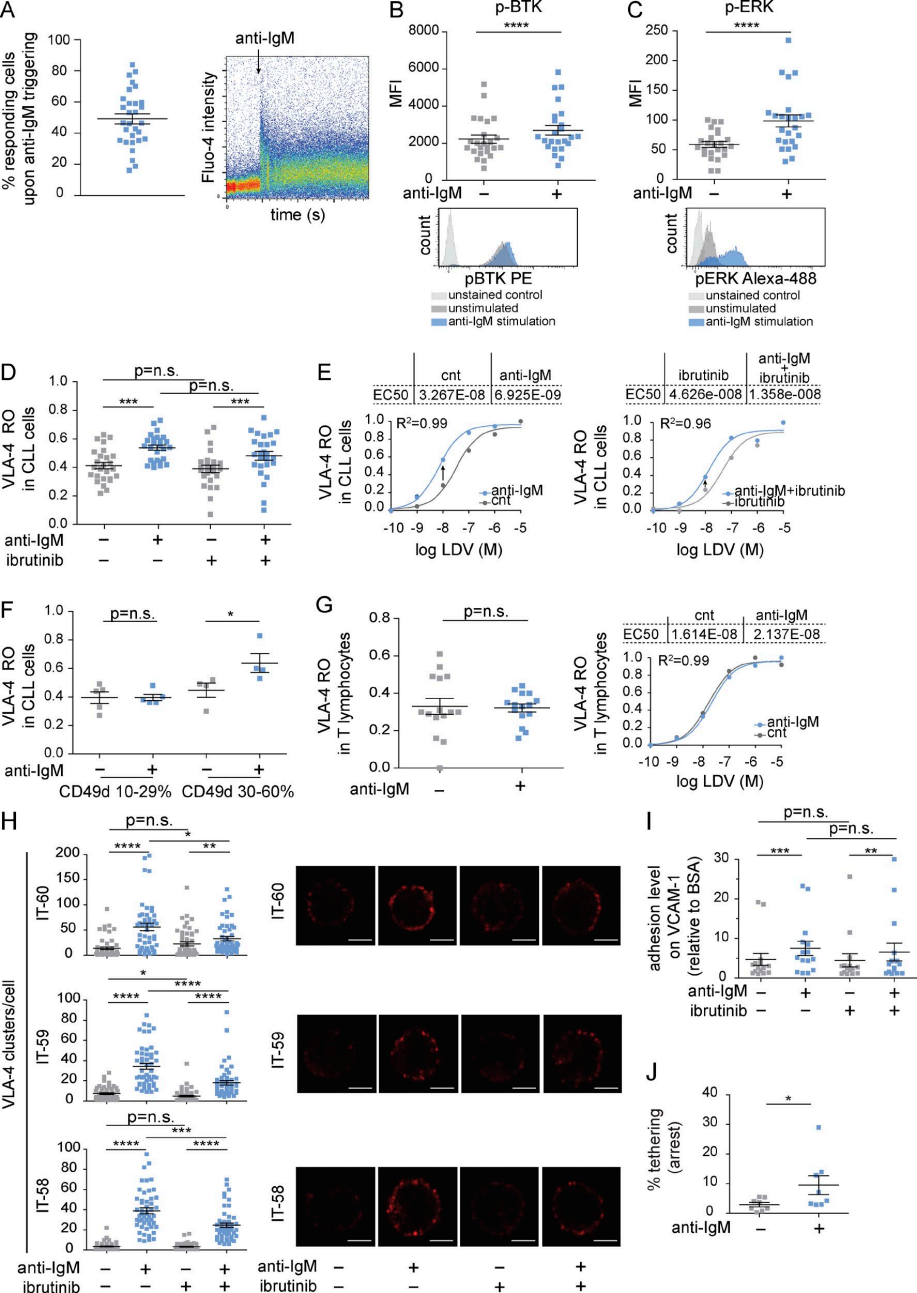
**Triggering of the BCR by anti-IgM induces VLA-4 activation and increases cell adhesion and VLA-4 clustering in CLL cells also upon ibrutinib treatment in vitro. (A)** Calcium response to anti-IgM stimulation in PBMCs from 30 CLL cases. The pseudocolored dot plot on the right shows an example of BCR response time (in seconds) over Fluo-4 intensity in one representative CLL case. Addition of the stimulus is indicated by the arrow. **(B and C)** MFIs of phospho (p)-BTK (B) and p-ERK (C) in unstimulated and anti-IgM stimulated cells from 24 CLL cases. The histogram plot overlays below the graphs show p-BTK (B) and p-ERK (C) expression in the unstimulated and anti-IgM–stimulated cells from one representative CLL case; the light gray histograms correspond to unstained cells. **(D)** VLA-4 RO in primary CLL cells from 26 cases (all cases expressing >60% CD49d), pretreated or not with 1 µM ibrutinib and stimulated or not with 5 µg/ml anti-IgM. The reported VLA-4 RO values were calculated as described in Materials and methods and correspond to the presence of 10 nM LDV. **(E)** VLA-4 RO plotted versus LDV concentration using the sigmoidal dose–response equation with variable slope performed by GraphPad Prism software in cells untreated (left) or treated with ibrutinib (right) in one representative CLL case. The arrows indicate the shift from the control to the anti-IgM–stimulated condition at 10 nM LDV. EC_50_ values for all conditions are indicated. R^2^ corresponds to the coefficient of determination. **(F)** VLA-4 RO in primary CLL cells expressing CD49d between 10% and 29% (*n* = 5) and between 30% and 60% (*n* = 4), pretreated or not with 1 µM ibrutinib and stimulated or not with 5 µg/ml anti-IgM. **(G)** VLA-4 RO in T lymphocytes from 15 CLL cases in unstimulated and anti-IgM–stimulated conditions. The line graph on the right shows the VLA-4 RO plotted versus LDV concentration obtained in T-lymphocytes from one representative CLL case. **(H)** VLA-4 clusters in primary CLL cells from 3 cases treated as in (D) obtained on VCAM-1-coated slides. Quantitative clustering analysis was done in at least 50 individual cells for each condition by means of confocal microscopy (original magnification ×60). The three dot plots correspond to three different cases and show the cluster number for all analyzed cells in each condition. On the right, representative confocal microscopy images of VLA-4 clusters revealed by anti-CD49d mAbs, are shown. Bars, 2 µm. **(I)** Adhesion on VCAM-1 of primary CLL cells from 15 cases treated as in D. Cell adhesion was calculated as relative fold change obtained on VCAM-1 over BSA. Each experiment was run in triplicate. **(J)** Purified CLL cells from eight cases were stimulated or not with anti-IgM and perfused for 1 min at 0.5 dyn/cm^2^ over immobilized VCAM-1. The data are expressed as the mean of frequencies of cells in direct contact with the substrate (tethering). Each experiment was run in triplicate. Data are presented as mean ± SEM. Individual symbols represent individual cases in all panels except H, which represents individual cells. *, P < 0.05; **, P < 0.01; ***, P < 0.001; ****, P < 0.0001; n.s., not significant (Wilcoxon test in all panels except F, where a paired t test was applied).

The capability of the VLA-4 integrin to be inside-out activated by stimuli originating from the BCR (Spaargaren et al., 2003; Arana et al., 2008a) was tested in the context of CLL cells. For this purpose, we first evaluated the anti-IgM–induced BCR response in ibrutinib-naive cells from 30 CLL cases, all expressing CD49d above the established cutoff of 30% of positive cells (Table S1; Gattei et al., 2008). BCR signaling response was measured by analyzing the anti-IgM–induced calcium mobilization by flow cytometry. In all cases, the cells responded to BCR triggering according to the 5% cutoff of responding cells (Fig. 1 A; Mockridge et al., 2007), although with variability. Similarly, CLL cells stimulated with anti-IgM also variably increased the phosphorylation levels of BTK and extracellular signal-regulated kinases (ERKs; Fig. 1, B and C). The virtual absence of nonresponder cases in this CLL cohort may be explained by the correlation of high CD49d expression with the presence of other negative prognostic factors, such an unmutated (UM) immunoglobulin heavy chain variable *(IGHV)* mutational status and *TP53* disruption (Table S1), which have previously been shown to be associated with BCR responsiveness (Mockridge et al., 2007; Apollonio et al., 2013).

We next studied in the same CLL cases the effects of BCR stimulation on VLA-4 activation by measuring VLA-4 affinity by flow cytometry using a peptide ligand derived from the LDV sequence of the VLA-4–binding region of fibronectin (hereafter LDV) as VLA-4 specific ligand, along with the conformation sensitive anti–CD29 HUTS-21 mAb, to measure the VLA-4 receptor occupancy (RO) in values ranging from 0.0 (no RO) to 1.0 (100% RO) as previously described (Chigaev et al., 2009). BCR triggering significantly increased VLA-4 RO, when probed at fixed ligand concentration (Fig. 1 D), as documented by a significant shift of the binding curve toward lower LDV ligand concentration, clearly indicating higher ligand-binding affinity (Fig. 1 E, left). The BCR-induced VLA-4 activation was not restricted to anti-IgM stimuli because anti-IgD was also able to trigger the BCR signaling cascade (Fig. S1 A; Ten Hacken et al., 2016) and efficiently elicit VLA-4 activation responses (Fig. S1 A). Similar results were obtained with normal B cells from the peripheral blood (PB) of healthy volunteers (*n* = 9), where anti-IgM triggering was also able to induce a BCR response in terms of phospho (p)-BTK and p-AKT (Fig. S1 B). Moreover, normal B cells, all expressing both CD29 and CD49d at high levels, increased VLA-4 activation upon anti-IgM stimuli (Fig. S1 B).

With the aim to establish the CD49d minimum expression levels that enable activation by anti-IgM, an additional set of experiments were performed using CLL cells from CD49d^−^ cases (i.e., expressing CD49d below the 30% clinical cutoff). In this context, VLA-4 RO could not be calculated for CLL cases with CD49d expression <10% (*n* = 4; not depicted) because these cells showed no detectable dose-dependent increase in the exposure of the ligand-induced epitope recognized by HUTS-21 (see Materials and methods for details). On the contrary, samples with CD49d expression between 10% and 29% (i.e., still formally negative) showed a dose-dependent increase in the exposure of the ligand-induced epitope recognized by HUTS-21, but no activation by anti-IgM could be observed (Fig. 1 F). Of note, increased VLA-4 activation upon anti-IgM triggering could be experimentally appreciated with CD49d expression levels between 30% and 60%, corresponding to intermediate levels of expression, thus corroborating the use of the 30% CD49d clinical cutoff in this setting (Fig. 1 F). The VLA-4 affinity change upon BCR triggering was specific to CLL cells because no differences in VLA-4 activation were observed in the residual T cell population, whose VLA-4 activation levels remained unaffected by BCR stimulation (Fig. 1 G).

Consistently, evaluation of integrin clustering by immunofluorescence analysis showed enhanced VLA-4 clustering after anti-IgM stimulation (Fig. 1 H), reflecting higher levels of avidity to the ligand (Spaargaren et al., 2003).

The VLA-4 activation correlated with the increased capacity of purified CLL cells to adhere on VCAM-1 upon BCR stimulation (Fig. 1 I). Adhesion data were confirmed by a dynamic shear flow assay on VCAM-1, where the percentage of arresting cells increased under anti-IgM stimulation (Fig. 1 J).

### BCR-mediated VLA-4 activation is retained upon ibrutinib treatment in vitro

The effects of ibrutinib-mediated BCR inhibition on VLA-4 activation were then investigated in vitro using cells from the same CD49d^+^ CLL cases described above (Table S1). In keeping with previous studies (Herman et al., 2014a; Woyach et al., 2014b), 1 µM ibrutinib treatment impaired both constitutive and anti-IgM–stimulated BCR signaling, as witnessed by the significantly lower phosphorylation levels of BTK and ERK (Fig. S1 C) and of phospholipase C γ2 (PLCγ2) and AKT; these latter were evaluated in a smaller cohort of cases (*n* = 10; Fig. S1 C). Moreover, consistent with a previous study (Woyach et al., 2014a), ibrutinib treatment decreased the anti-IgM induced calcium release (Fig. S1 C). The impairment of the BCR-related signaling under ibrutinib was also evident when comparing the phosphorylation levels of BTK and the immediate downstream PLCγ2, which did not significantly differ between the anti-IgM and the unstimulated conditions under ibrutinib. However, we observed a residual activity of both AKT and ERK, whose phosphorylation levels were found to increase upon anti-IgM triggering also under ibrutinib (Fig. S1 C).

Despite the overall lower BCR signaling caused by CLL exposure to ibrutinib, ibrutinib-treated CLL cells unexpectedly retained their capability to activate VLA-4 upon anti-IgM triggering. Indeed, the VLA-4 RO values measured after anti-IgM stimulation increased significantly, reaching levels comparable with those observed in ibrutinib-naive cells (Fig. 1 D), and the binding curve shifted accordingly (Fig. 1 E, right). Moreover, despite an overall impairment of the anti-IgM–induced VLA-4 clustering upon ibrutinib treatment compared with the untreated condition, BCR triggering was still able to induce VLA-4 clusters in the context of ibrutinib-treated cells, suggesting that the integrin avidity for the ligand is only partially affected by BTK inhibition (Fig. 1 H).

In keeping with the retention of VLA-4 activation, CLL cells treated in vitro with ibrutinib were still able to increase their adhesion levels on VCAM-1 upon anti-IgM stimulation to an extent similar to that observed in ibrutinib-naive cells (Fig. 1 I).

### BCR-mediated VLA-4 activation is retained in ibrutinib-treated CLL cells in vivo

Serial samples from CLL patients treated in vivo with ibrutinib were collected at baseline and at days 7, 30, and 90 after the initiation of ibrutinib therapy.

During the course of the therapy, a progressive impairment of BCR signaling was observed, as previously reported (Herman et al., 2014a; Woyach et al., 2014b). The constitutive phosphorylation levels of BTK, ERK, PLCγ2, and AKT pretreatment decreased over time, reaching a significant difference at day 90 (Fig. S1 D). Moreover, anti-IgM–triggered BCR signaling showed a progressive decrease from pretreatment to day 90 in terms of calcium release and of BTK and PLCγ2 phosphorylation, these last reaching phosphorylation levels at day 90 often comparable to their corresponding unstimulated conditions (Fig. S1 D). On the contrary, only a mild reduction of p-AKT and p-ERK was documented during the course of ibrutinib therapy, reaching the statistical significance compared with day 0 only in the case of p-AKT levels at day 90 (Fig. S1 D).

**Figure 2.**
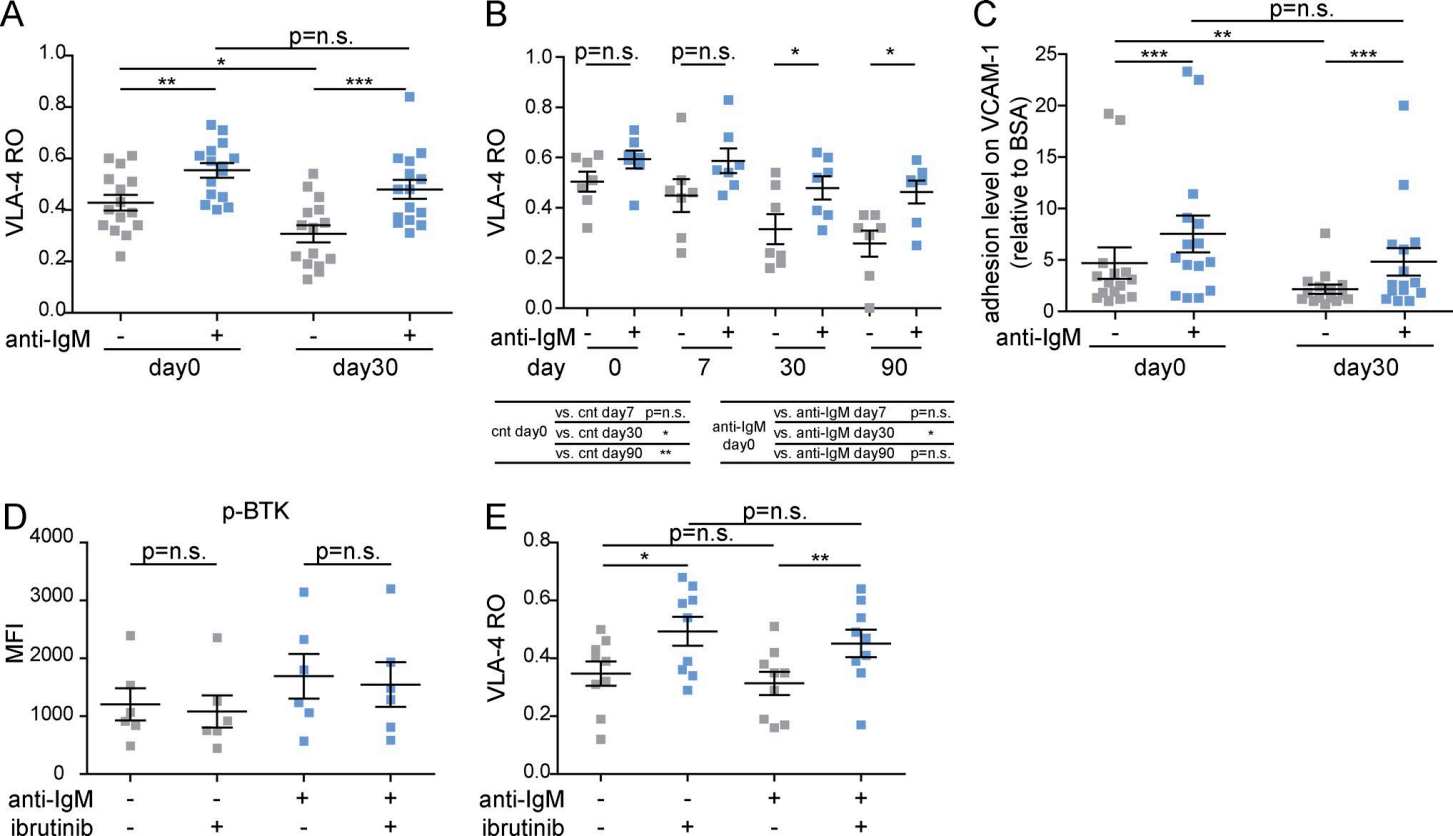
**BCR-mediated VLA-4 integrin activity is not affected by ibrutinib treatment in vivo.** Serial samples from 9 CLL patients were obtained before treatment (day 0) and at days 7, 30, and 90 on ibrutinib therapy. **(A)** VLA-4 RO in unstimulated and anti-IgM–stimulated CLL cells at days 0 and 30 on ibrutinib (*n* = 15). **(B)** VLA-4 RO in unstimulated and anti-IgM–stimulated CLL cells at days 0, 7, 30, and 90 on ibrutinib (*n* = 7). **(C)** Adhesion values on VCAM-1 in unstimulated and anti-IgM–stimulated cells at days 0 and 30 on ibrutinib. Each adhesion experiment was run in triplicate. **(D)** Phospho (p)-BTK MFI in unstimulated and in anti-IgM–stimulated conditions obtained in CLL cells collected at day 30 of ibrutinib therapy after incubation or not in vitro with 1 µM ibrutinib for 1 h (*n* = 8). **(E)** VLA-4 RO in the control condition and in anti-IgM–stimulated cells in CLL cells treated as in D. Data are presented as mean ± SEM. Individual symbols represent individual cases. *, P < 0.05; **, P < 0.01; ***, P < 0.001; n.s., not significant (Wilcoxon test).

We then analyzed the impact of ibrutinib therapy on both constitutive and anti-IgM–stimulated VLA-4 activation as determined by measuring the RO values corresponding to the 10 nM LDV concentration. The levels of constitutive VLA-4 activation after 30 d of ibrutinib treatment were significantly lower than pretreatment levels (*n* = 15; Fig. 2 A). Similarly, in a subgroup of CLL with samples available before treatment and at days 7, 30, and 90 on ibrutinib (*n* = 7), constitutive VLA-4 activation levels observed before treatment progressively decreased starting from day 7 and reached lower levels at days 30 and 90 (Fig. 2 B). Conversely, BCR triggering by anti-IgM significantly increased VLA-4 activation both in CLL cells before treatment and in CLL cells collected at day 30 of ibrutinib therapy (Fig. 2 A). Notably, BCR triggering still induced VLA-4 activation after 90 d of ibrutinib treatment to levels comparable with those observed before treatment (Fig. 2 B).

We next determined the effects of in vivo ibrutinib treatment on CLL cell adhesion on VCAM-1. CLL cells obtained at day 30 of ibrutinib showed significantly reduced adhesion levels compared with pretreatment cells. Moreover, in line with the VLA-4 activation data, the ability of BCR triggering to increase VLA-4–mediated CLL cell adhesion observed before treatment was also maintained in CLL cells collected at day 30 of treatment, with no significant difference compared with the pretreatment condition (Fig. 2 C).

To rule out the possibility of an incomplete BTK inhibition in the in vivo ibrutinib-treated CLL cells used in our experiments, both constitutive and anti-IgM–stimulated BTK phosphorylation and VLA-4 activation were analyzed after the addition of an excess of 1 µM ibrutinib to CLL cells collected at day 30 of ibrutinib therapy (*n* = 8). In vitro, the addition of ibrutinib did not further inhibit the constitutive and anti-IgM–stimulated phosphorylation of BTK (Fig. 2 D), and no difference was found in either constitutive or anti-IgM–stimulated VLA-4 activation levels after ibrutinib addition (Fig. 2 E).

Moreover, to definitely exclude the possibility that the observed BCR-induced VLA-4 activation characterizing CLL cells on ibrutinib treatment was caused by loss of BTK inhibition, CLL cells were tested by next-generation sequencing (NGS) for the presence of *BTK* and *PLCγ2* mutations, which have been reported to confer ibrutinib resistance (Woyach et al., 2014a). Analysis of C418S *BTK* mutations and R665W, S707Y, and L845F *PLCγ2* mutations in CLL cells collected at day 30 of ibrutinib excluded the presence of acquired mutations in all samples (*n* = 8; Table S1).

### Concomitant inhibition of BTK and PI3K impairs VLA-4 activation

**Figure 3.**
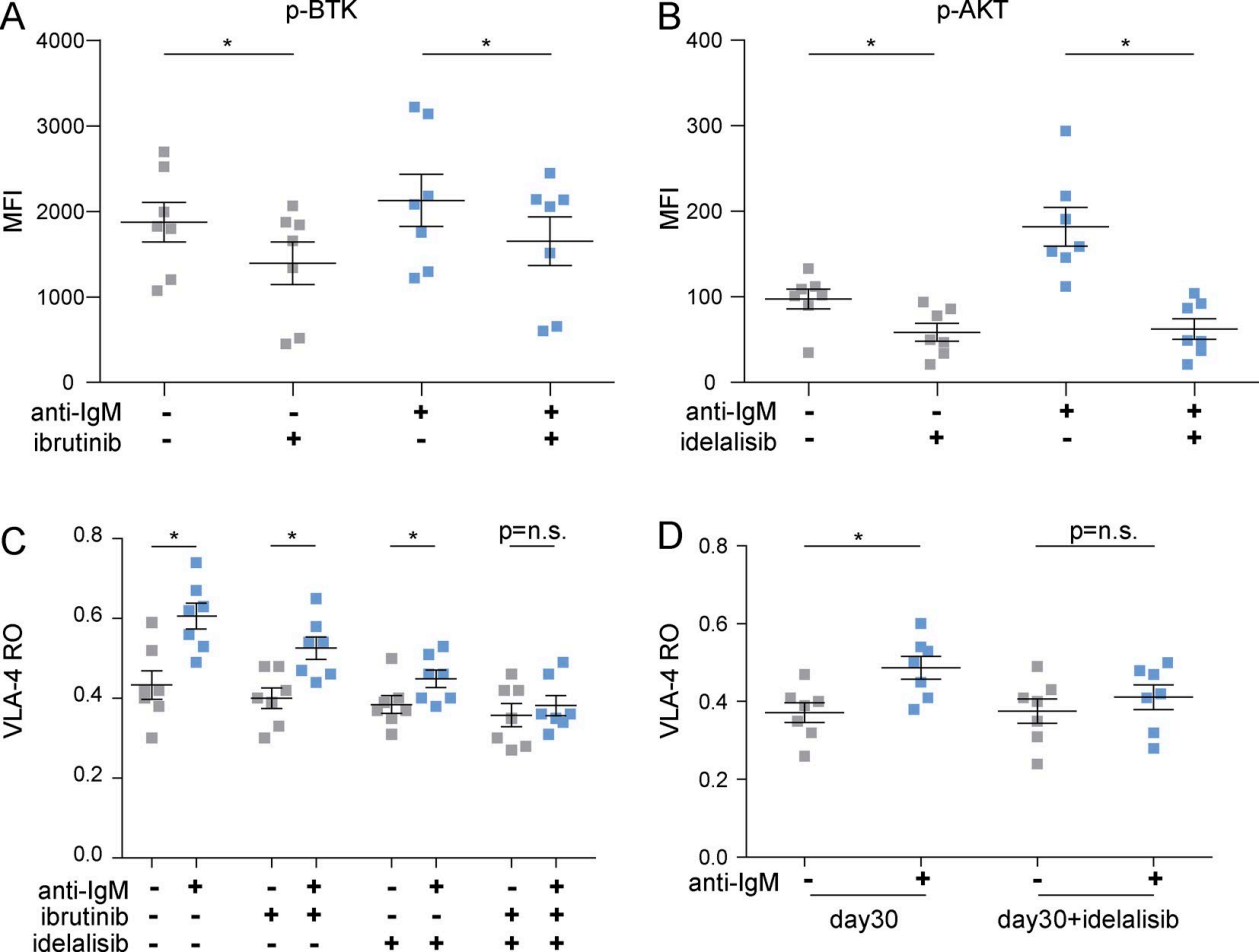
**Concomitant inhibition of BTK and PI3K impairs VLA-4 activation. (A)** Phospho (p)-BTK MFI in unstimulated and anti-IgM–stimulated CLL cells from seven cases pretreated or not with 1 µM ibrutinib. **(B)** p-AKT MFI in unstimulated and anti-IgM–stimulated CLL cells pretreated or not with 1 µM idelalisib. **(C)** VLA-4 RO in unstimulated and anti-IgM–stimulated CLL cells untreated or treated with 1 µM ibrutinib, 1 µM idelalisib, or a combination of both. **(D)** VLA-4 RO in unstimulated and anti-IgM–stimulated CLL cells (*n* = 7) collected at day 30 of ibrutinib therapy and treated or not with 1 µM idelalisib in vitro. Data are presented as mean ± SEM. Individual symbols represent individual cases. *, P < 0.05; n.s., not significant (Wilcoxon test).

The data reported so far suggest that BTK inhibition is not sufficient to block BCR-dependent VLA-4 activation. Indeed, BCR-induced inside-out activation of another integrin, LFA-1, has been reported to occur through a BTK-independent pathway involving phosphatidylinositide 3-kinase (PI3K; Arana et al., 2008a). Because residual phosphorylation of the downstream PI3K target AKT was observed in ibrutinib-treated CLL cells upon BCR stimulation (Fig. S1, C and D), we next investigated the effects of combined BTK and PI3K inhibition on BCR-induced VLA-4 activation. For this purpose, we treated CLL cells from 7 cases with 1 µM ibrutinib, 1 µM idelalisib, or a combination of both. A significant decrease in BTK and AKT phosphorylation levels confirmed the actual inhibition of the BCR pathway driven by ibrutinib and idelalisib, respectively (Fig. 3, A and B). Analysis of VLA-4 activation showed that neither ibrutinib nor idelalisib, when used as single agents, was able to fully block the anti-IgM–induced VLA-4 activation, whereas a concomitant inhibition of both BTK and PI3K completely abolished the integrin response to BCR triggering (Fig. 3 C). These data were confirmed in ibrutinib-treated CLL cells (*n* = 7), where the addition of idelalisib to CLL cells collected at day 30 on ibrutinib was able to completely antagonize anti-IgM–induced VLA-4 activation (Fig. 3 D). The key role of both BTK and PI3K in the induction of VLA-4 activation was also observed in normal B cells from healthy donors (Fig. S1 B), where, similarly to what we reported in CLL cells, only concomitant inhibition of both BTK and PI3K was able to abrogate anti-IgM–induced VLA-4 activation.

### CD49d^+^ CLL displays reduced ibrutinib-induced redistribution lymphocytosis

**Figure 4.**
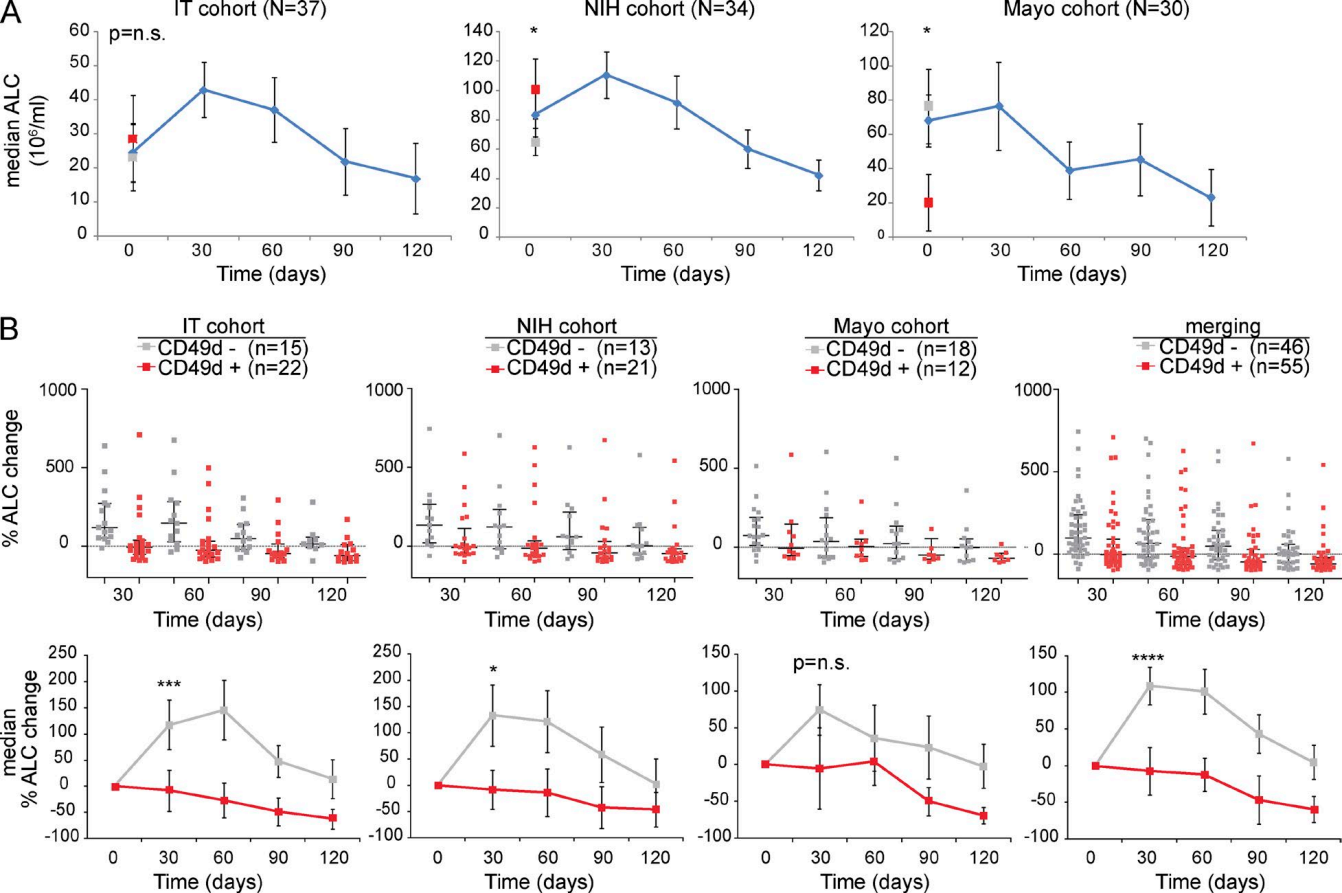
**CD49d^+^ and CD49d^−^ CLL show different patterns of redistribution lymphocytosis during ibrutinib treatment.** ALCs were collected pretreatment (day 0) and at different treatment time points (days 30, 60, 90, 120) in CLL cases from three different cohorts. **(A)** Kinetics of ALC (median values) in the IT cohort (left), NIH cohort (middle), and Mayo cohort (right); the gray and red symbols in each graph correspond to the pretreatment median ALC in CD49d^−^ and CD49d^+^ CLL, respectively. **(B)** Percent ALC change from baseline in CLL cases split according to CD49d expression (CD49d^+^, red; CD49d^−^, gray) from the three individual cohorts and the merging of all cohorts, shown as individual plots (above graphs) and as median values (below graphs). The number of patients included in each group are reported in parentheses; the black vertical lines indicate SEM. *, P < 0.05; ***, P < 0.001; ****, P < 0.0001; n.s., not significant (Mann–Whitney test).

Absolute lymphocyte count (ALC) data were collected before treatment and at days 30, 60, 90, and 120 on ibrutinib from three independent cohorts of patients receiving ibrutinib as single agent (one from Italy [*n* = 37, hereafter IT cohort] and two from the United States: National Institutes of Health, Bethesda, MD [*n* = 34, hereafter NIH cohort] and Mayo Clinic, Rochester, MN [*n* = 30, hereafter Mayo cohort]; Materials and methods; Table S1). The median baseline ALCs were 24.7 × 10^6^/ml, 83.6 × 10^6^/ml, and 68.0 × 10^6^/ml for the IT, NIH, and Mayo cohorts, respectively. Despite these differences, similar trends of ALC kinetics, peaking at day 30 of ibrutinib treatment, were found in the three cohorts (Fig. 4 A).

We then compared ALC kinetics in CD49d^−^ and CD49d^+^ CLL using the percent ALC change to normalize for the differences in ALC absolute values. Heterogeneous baseline levels of ALC were found in the different cohorts irrespective of CD49d expression (Fig. 4 A) and, as expected (Herman et al., 2014b; Farooqui et al., 2015; Thompson et al., 2015a), variability in ALC values was observed in both CD49d^−^ and CD49d^+^ CLL cases upon ibrutinib treatment (Fig. 4 B, top). This notwithstanding, in all three cohorts analyzed separately or merged together, CD49d^+^ CLL mostly failed to display an increase of the median ALC values at the analyzed time points, whereas the typical ibrutinib-induced early peak of ALC values was observed in CD49d^−^ CLL (Fig. 4 B, bottom). When considering the later time points at days 60, 90, and 120, the trend of percent ALC change in the CD49d^+^ CLL group was again similar among the three different cohorts (Fig. 4 B, bottom).

The different trends in lymphocytosis from pretreatment to day 30 between CD49d^−^ and CD49d^+^ CLL were also observed in a small group of patients (*n* = 10) of the Mayo cohort, entering the clinical trial NCT02048813 which introduced rituximab after 30 d of ibrutinib (Materials and methods; Table S1). As expected, the addition of rituximab at day 30 resulted in a rapid decrease in ALC values in CD49d^−^ CLL, which became superimposable to those of CD49d^+^ CLL at later time points (Fig. S2 A).

**Figure 5.**
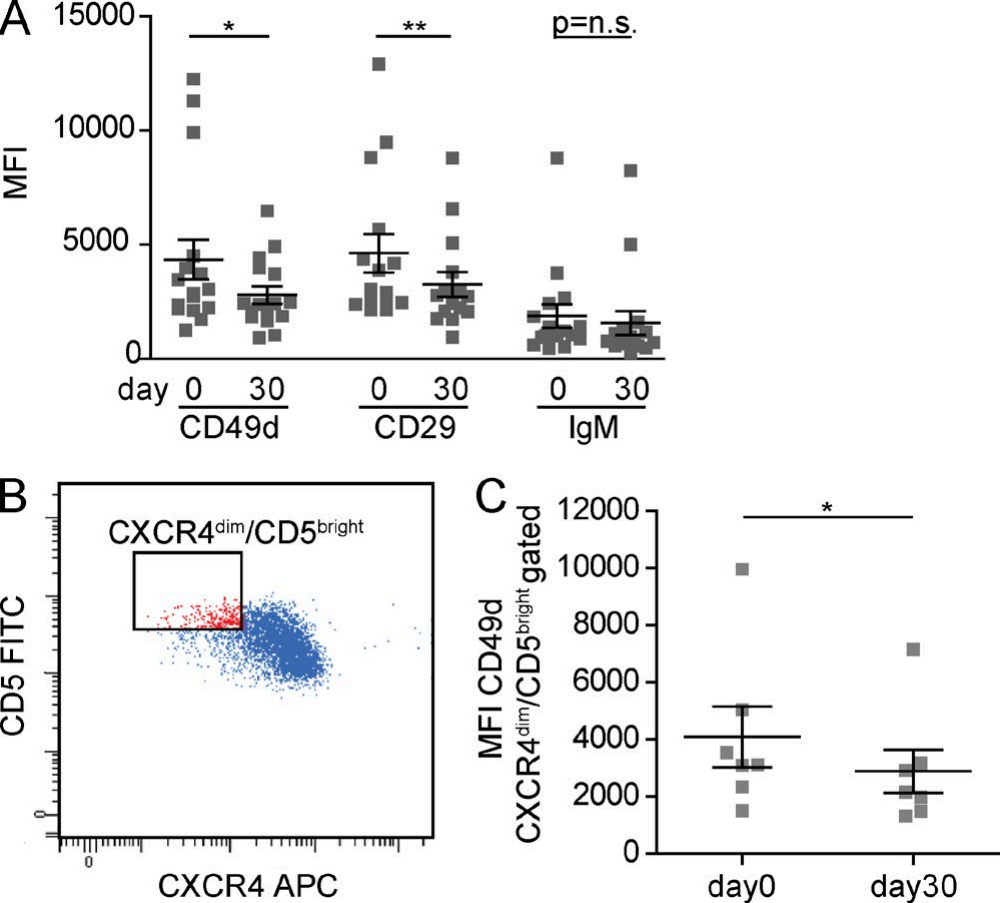
**VLA-4 expression decreases after ibrutinib therapy. (A)** Flow-cytometric determination of CD49d, CD29, and IgM MFI in CLL cells collected at days 0 and 30 on ibrutinib. **(B)** Representative flow cytometry dot plot showing CXCR4 versus CD5 expression in gated CLL cells. The red gate identifies CXCR4^dim^/CD5^bright^ population. **(C)** CD49d MFI in the CXCR4^dim^/CD5^bright^ population of CLL cells collected at days 0 and 30 on ibrutinib. Data are presented as mean ± SEM. Individual symbols represent individual cases. *, P < 0.05; **, P < 0.01; n.s., not significant (Wilcoxon test).

Given the influence of CD49d expression on ibrutinib-induced lymphocytosis, we next evaluated its expression in PB CLL cells collected at baseline and after 30 d of treatment. A decrease of expression of both VLA-4 subunits (CD49d/CD29) was observed at day 30 (Herman et al., 2015), without reduction of IgM expression (Fig. 5 A). Notably, such a decrease occurred also in the context of the CXCR4^dim^/CD5^bright^ population, which has been reported to represent the proliferative fraction, highly enriched in cells that have just been released from lymphoid tissues, where activation and proliferation occur (Fig. 5, B and C; Calissano et al., 2011).

### CD49d^+^ CLL displays reduced ibrutinib-induced LN shrinkage

**Figure 6.**
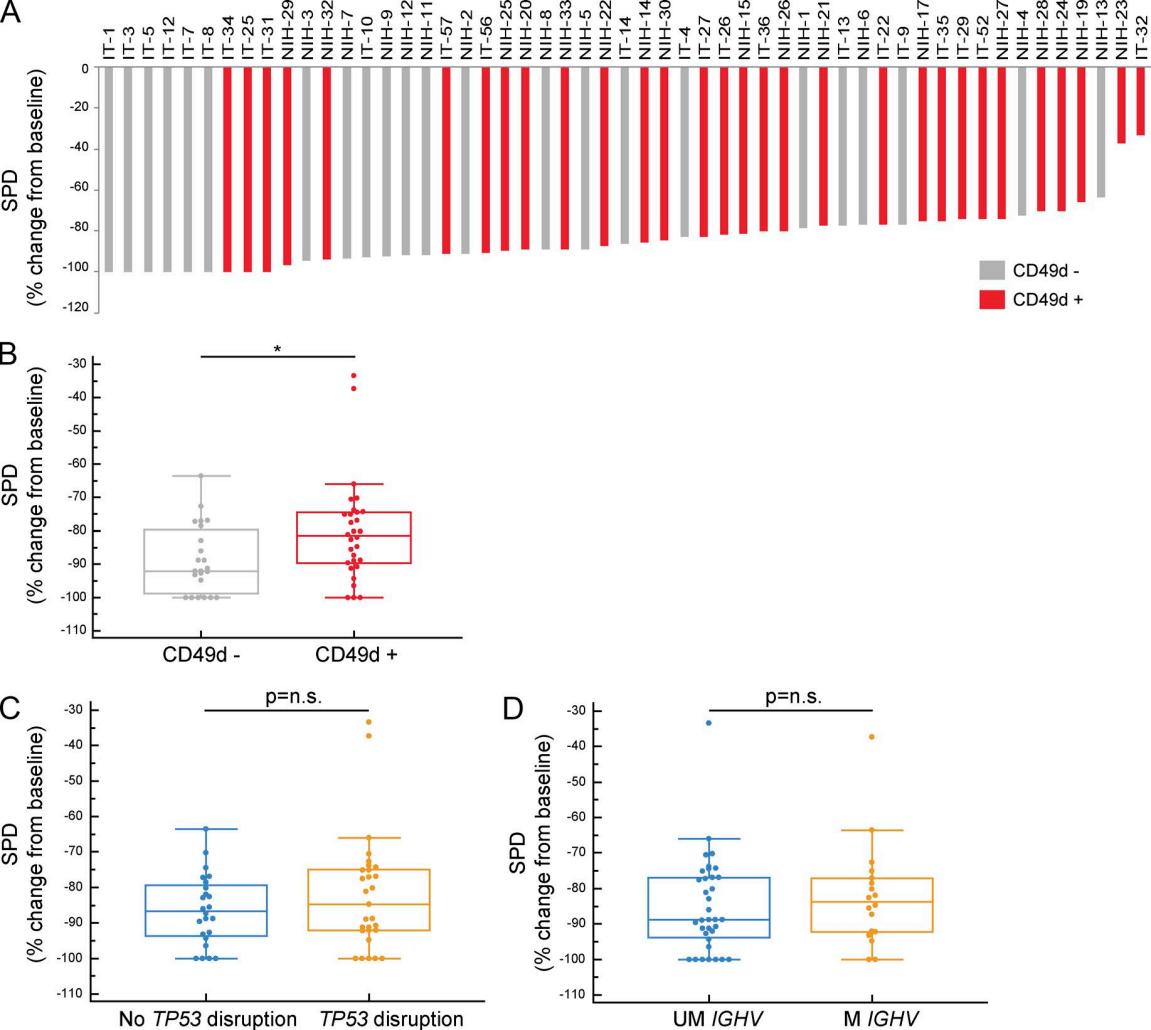
**CD49d^+^ and CD49d^−^ CLL are characterized by different ibrutinib-induced nodal responses.** LN dimension evaluation was performed pretreatment and at 12 mo of ibrutinib therapy, and the sum of the products of LN diameters (SPD) of up to five LN regions was calculated. **(A)** Waterfall plot showing the percent SPD change from baseline in CD49d^+^ (red bars) or CD49d^−^ (gray bars) CLL cases. **(B–D)** The box and whisker plot shows the median percent SPD change in CLL cases split according to CD49d expression (CD49d^−^, *n* = 23; gray box; CD49d^+^, *n* = 30; red box; B), *TP53* disruption (no *TP53* disruption, *n* = 24; blue box; *TP53* disruption, *n* = 28; orange box; C), and *IGHV* mutations (UM, *IGHV*, *n* = 35; blue box; mutated [M] *IGHV*, *n* = 18; orange box; D). *, P < 0.05; n.s., not significant (Mann–Whitney test).

We then evaluated the impact of CD49d expression on the extent of lymphadenopathy reduction induced by ibrutinib therapy. Sum of the product of diameter (SPD) values of the major LN regions were available before treatment and at 2, 6, or 12 mo on therapy (Table S1). Nodal response was calculated as percent reduction in SPD values, as reported previously (Byrd et al., 2013). Overall, the median LN reduction was 54.8% at 2 mo (*n* = 33, NIH cohort), 76.2% at 6 mo (*n* = 33, NIH cohort), and 86.1% at 12 mo (*n* = 53, NIH and IT cohorts; Fig. S2 B). We observed a trend toward lower median LN reduction in CD49d^+^ CLL compared with CD49d^−^ CLL at 2 mo (CD49d^+^ CLL, *n* = 20; CD49d^−^ CLL, *n* = 13; Fig. S2 C, top), which manifested in a significant effect at both 6 mo (CD49d^+^ CLL, *n* = 20; CD49d^−^ CLL, *n* = 13; Fig. S2 C, bottom) and 12 mo (CD49d^+^ CLL, *n* = 30; CD49d^−^ CLL, *n* = 23; Fig. 6, A and B). On the contrary, the degree of nodal response was not correlated with other prognostic markers, including *TP53* disruption, defined by the presence of either *TP53* mutation or 17p−, and *IGHV* mutational status (Fig. 6, C and D).

Correlation analyses between the levels of CD49d expression, defined as percentage of positive cells (all cohorts) or mean fluorescence intensity (MFI) values (IT cohort), and the ibrutinib-induced responses (i.e., ALC change at day 30 and percent SPD change at 12 mo), where the largest number of cases were available, highlighted a significant relationship that was mainly driven by the negative/positive expression of CD49d (i.e., below or above the 30% cutoff; Fig. S3, A–C). This observation is in line with the functional data on VLA-4 activation, where the 30% CD49d expression value roughly corresponded to the threshold amount of VLA-4 molecules that has to be present on the cell surface to detect an effective inside-out activation by BCR triggering (see above; Fig. 1 F).

### CD49d expression is correlated with shorter PFS under ibrutinib

At the median follow up of 24.5 mo, 28 (27.7%) of 101 patients progressed or died on study.

An univariate analysis was performed testing the prognostic relevance as PFS predictors of CD49d expression (CD49d^+^, *n* = 55) and other biological and clinical biomarkers (Table S1), including age (>65 yr, *n* = 54), gender (males, *n* = 60), Rai stage (III–IV Rai stage, *n* = 55), treatment-naive (TN)/refractory-relapsed (RR) status (RR cases, *n* = 69), white blood cell count, β2 microglobulin (≥ 4 mg/l, *n* = 37), *IGHV* gene mutational status (UM *IGHV*, *n =* 67), *TP53* disruption (*TP53* disrupted, *n* = 42), and 11q deletion (del11q, *n* = 19).

**Figure 7.**
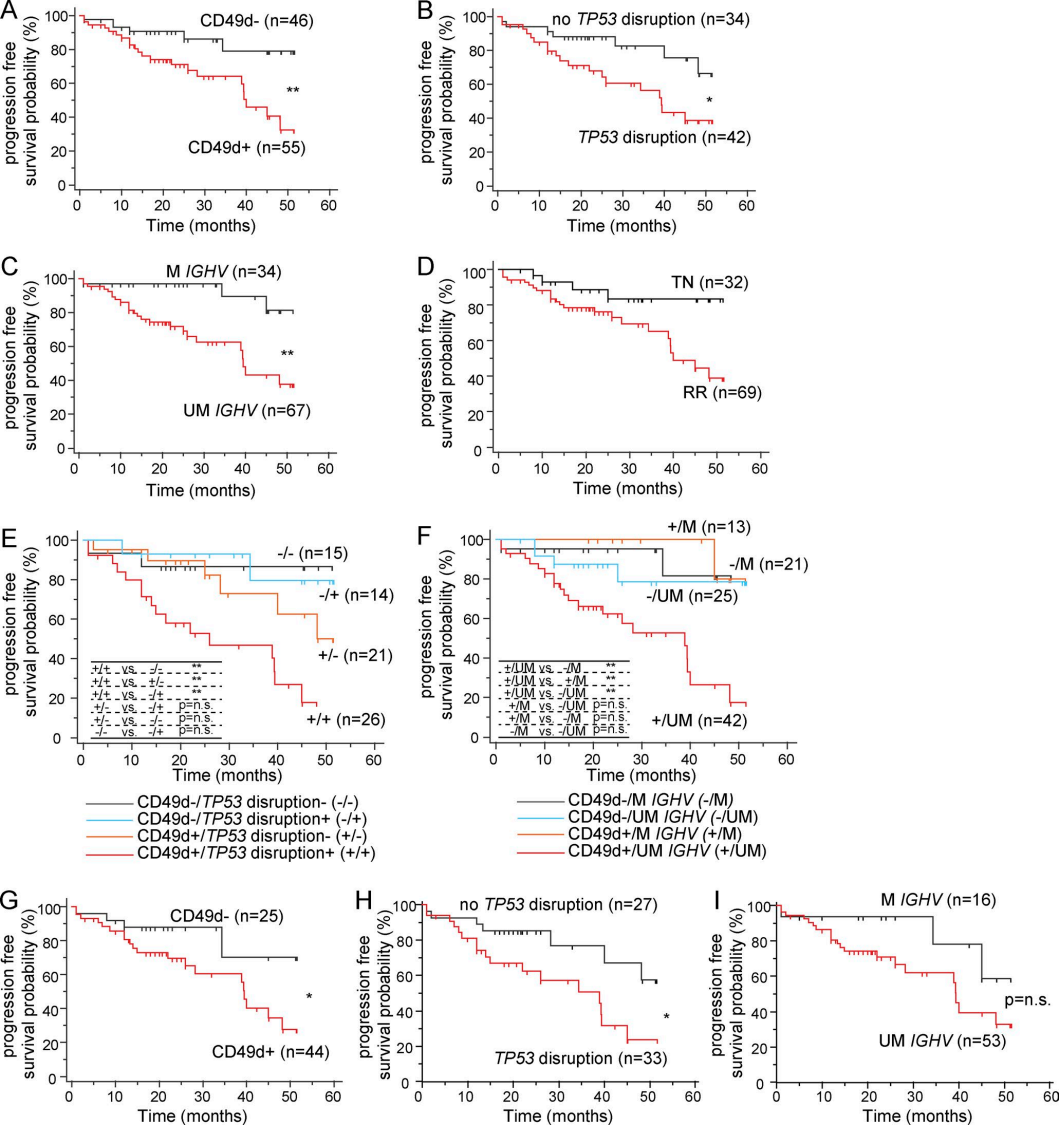
**PFS of ibrutinib-treated CLL patients. (A-I)** Kaplan–Meier estimates of PFS of CLL patients treated with ibrutinib and stratified by CD49d expression (A), *TP53* disruption (B), *IGHV* mutational status (C), prior treatment (D), the association of CD49d expression and *TP53* disruption (E), the association of CD49d expression and *IGHV* mutational status (F), CD49d expression in the context of RR CLL (G), *TP53* disruption in the context of RR CLL (H), and *IGHV* mutational status in the context of RR CLL (I). The number of patients included in each group are reported in parentheses. *, P < 0.05; **, P < 0.01; n.s., not significant (log-rank test).

Significantly inferior PFS was observed with CD49d^+^ CLL compared with CD49d^−^ CLL (Fig. 7 A and Table 1), whereas among the other variables, only *TP53* disruption, *IGHV* gene mutations, and the TN/RR status predicted shorter PFS, in keeping with previously reported observations (Byrd et al., 2015; Ahn et al., 2017; Fig. 7, B–D; and Table 1). The lack of impact of the other baseline characteristics on PFS is consistent to what was observed in the ibrutinib arm of the stage 3 RESONATE study or other studies of real-world experience with ibrutinib (Winqvist et al., 2016; Brown et al., 2017).

**Table 1.** Univariate and multivariate Cox regression analysis of PFS in ibrutinib-treated CLL

Analysis	HR (95% CI)	P
**Univariate analysis**		
Age ≥65 yr	0.48 (0.22–1.05)	0.068
Sex, male	0.50 (0.24–1.06)	0.071
III–IV Rai stage	1.30 (0.56–3.03)	0.548
Therapy, RR status	3.14 (1.09–9.01)	0.034
WBC count	1.00 (1.00–1.00)	0.364
β2M ≥4 mg/L	1.06 (0.43–2.62)	0.894
CD49d^+^	3.32 (1.35–8.17)	0.009
UM *IGHV*	5.48 (1.65–18.17)	0.006
* TP53* disrupted	2.71 (1.14–6.43)	0.025
Del11q	0.52 (0.16–1.74)	0.291
**Multivariate analysis**		
Model 1		
CD49d^+^	3.15 (1.16–8.53)	0.025
UM *IGHV*	4.17 (1.23–14.15)	0.023
* TP53* disrupted	2.83 (1.18–6.82)	0.021
Model 2		
CD49d^+^	2.61 (1.03–6.61)	0.043
UM *IGHV*	4.32 (1.26–14.81)	0.020
Therapy, RR status	1.53 (0.50–4.65)	0.457
Model 3		
CD49d^+^	2.61 (0.95–7.16)	0.065
* TP53* disrupted	3.00 (1.24–7.29)	0.016
Therapy, RR status	3.71 (0.83–16.59)	0.087

White blood cell count was considered as continuous variable. β2M, β2 microglobulin; CI, confidence interval; Del11q, according to Döhner et al. (2000); HR, hazard ratio; RR, relapsed-refractory; UM, unmutated; WBC, white blood cell.

Of note, the concomitant presence of CD49d^+^ expression and *TP53* disruption (Fig. 7 E) or UM *IGHV* (Fig. 7 F) predicted significantly inferior PFS compared with the presence of either factor alone.

When testing a multivariate Cox regression analysis, four variables were considered: CD49d expression, *TP53* status, *IGHV* gene status, and the TN/RR status. In our context, because of the low number of events, a multivariate analysis with more than three variables was not statistically recommended (Peduzzi et al., 1995). Therefore, a panel of multivariate analyses, which included all of the three-variable combinations of CD49d with the other main predictors, was performed. As summarized in Table 1, the results suggest that the three biological variables (i.e., CD49d, *TP53* disruption, and *IGHV* gene status) emerged as independent predictors in models including TN/RR status. Consistently, high CD49d expression, the presence of *TP53* disruption, and UM *IGHV* gene status (which did not reach statistical significance) identified a patient subset with shorter PFS intervals in the context of the RR cohort (Fig. 7, G–I).

## Discussion

The interaction between VLA-4 and the BCR has been described in B lymphocytes, where it contributes to antigen-specific B cell differentiation (Spaargaren et al., 2003; Arana et al., 2008a). Thus far, this potential interplay has not been investigated in the context of CLL. Using a conformationally sensitive anti-CD29 antibody and a small LDV-containing probe mimicking the VLA-4 ligands, we report that signaling through the BCR was sufficient to induce VLA-4 conformation changes resulting in enhanced affinity of the integrin (Chigaev et al., 2009). Moreover, BCR-induced inside-out signaling also resulted in increased clustering of VLA-4 molecules causing higher avidity, as previously reported in a model of nonhuman B cells (Spaargaren et al., 2003). This increased VLA-4 activity was reflected by the higher adhesion of the cells to VCAM-1 under static and shear-flow conditions.

A functional interplay between the BCR and VLA-4 in the CLL setting has been recently hypothesized following observations of inhibitory effects exerted by ibrutinib on VLA-4–mediated CLL cell adhesion (de Rooij et al., 2012; Herman et al., 2015). Here, by taking advantage of both in vitro and in vivo ibrutinib-treated CLL cells, we could confirm the relationship between ibrutinib exposure and impaired CLL cell adhesion on VCAM-1 substrates (which was more apparent in cells derived from in vivo treatment), with increased inhibition after 30 d of therapy. Consistently, during in-vivo ibrutinib treatment, we described a progressive reduction of constitutive VLA-4 activation that was paralleled by a diminished constitutive phosphorylation of BCR-related proteins. This inhibition of BCR signaling occurring in the absence of BCR cross-linking, which was previously observed (Herman et al., 2014a; Woyach et al., 2014b), suggests that BTK inhibition by ibrutinib by abrogation of antigen-independent cell-autonomous signaling (Dühren-von Minden et al., 2012) may also affect the constitutive VLA-4 activation state via inside-out mechanisms.

Unexpectedly, when triggering the BCR in CLL cells treated with ibrutinib either in vitro or in vivo, VLA-4 was still capable of being inside-out activated, as clearly determined by both a conformation change consistent with an increased affinity (Chigaev et al., 2009) and an increased capability to form clusters (Spaargaren et al., 2003). In keeping with the maintenance of this double functionality of the integrin activation process, VLA-4–expressing CLL cells retained the capability to bind VLA-4 substrates upon BCR engagement, even in the presence of ibrutinib. Although the present study represents the first to investigate the VLA-4 activation state in CLL, adhesion assays of CLL cells in the presence or absence of ibrutinib have been reported earlier (de Rooij et al., 2012; Herman et al., 2015). These studies, however, either did not address cellular adhesion upon BCR triggering (Herman et al., 2015) or lacked information about the VLA-4 (CD49d) expression state of CLL cases used for these adhesion assays (de Rooij et al., 2012).

The observation that BCR engagement was still able to induce VLA-4 activation and VLA-4-mediated adhesion in ibrutinib-treated CLL cells, as shown here, could have been caused by a total or partial lack of BTK inhibition in CLL cells. However, the following findings argue against this possibility: (a) the addition of an excess of ibrutinib in vitro to CLL cells derived from patients treated for 30 d with ibrutinib did not further inhibit the constitutive and anti-IgM–stimulated phosphorylation status of BTK and had no impact on the constitutive or anti-IgM–stimulated VLA-4 activation level; and (b) samples used in our experiments had functional BTK and showed no evidence of specific *BTK* or *PLCγ2* mutations, whose emergence has been described to confer resistance to ibrutinib (Woyach et al., 2014a; Liu et al., 2015; Ahn et al., 2017).

In our hands, ibrutinib effectively inhibited BCR signaling in terms of calcium release, BTK phosphorylation, and phosphorylation of PLCγ2, the immediate downstream target of BTK (Spaargaren et al., 2003), but exhibited partial inhibition of p-AKT and no inhibition of p-ERK (Rickert, 2013). A controversial impairment of AKT and ERK activation by BCR triggering in CLL cells of patients treated with ibrutinib for 9 mo has been reported (Woyach et al., 2014b) and was allegedly caused by anergic features of these samples, manifesting in a lack of responsiveness to BCR stimulation also at the pretreatment stage in many instances (Apollonio et al., 2013).

The precise signaling cascade underlying BCR-induced VLA-4 activation is far from being understood. The current knowledge derives from studies of normal B cell differentiation, where membrane-bound antigens, e.g., on the surface of follicular dendritic cells, can be presented via BCR to germinal center B cells, with subsequent inside-out VLA-4 activation that can facilitate the survival through VLA-4/VCAM-1 interactions, especially for those B cells expressing an appropriate BCR (Carrasco and Batista, 2006). In this context, BCR triggering can activate VLA-4 through an axis involving BTK, and eventually regulating VLA-4 avidity (Spaargaren et al., 2003). A parallel axis, involving PI3K and bypassing BTK has been reported (Arana et al., 2008b), although most of the knowledge on PI3K involvement in inside-out integrin activation mechanisms stems from studies on LFA-1 rather than VLA-4 integrin.

The maintenance of a certain degree of phosphorylation of AKT, a protein located downstream PI3K in the BCR signaling cascade, may suggest residual anti-IgM–induced BCR signaling activity that in turn may be responsible for the observed BCR-related VLA-4 activation that occurs during ibrutinib treatment. Indeed, addition of the PI3K inhibitor idelalisib to both in vitro and in vivo ibrutinib-treated CLL cells completely abolished anti-IgM–induced VLA-4 activation. Consistently, the concomitant inhibition of BTK and PI3K by ibrutinib and idelalisib, respectively, has been shown to hamper BCR-stimulated integrin-mediated CLL cell adhesion in a strong synergistic manner (de Rooij et al., 2015).

VLA-4 is also expressed at high levels in PB normal B cells of healthy donors (Möller et al., 1992). Moreover, we provide evidence that BCR stimulation can inside-out activate VLA-4 also in these cells and that concomitant inhibition of BTK and PI3K blocks such activation. This suggests that the mechanisms observed in CLL can be considered a remnant of activity occurring in normal B cells (e.g., during the antigen-dependent differentiation process occurring in lymphoid organs; Carrasco and Batista, 2006).

The variability in degree and kinetics of ibrutinib-induced recirculation lymphocytosis, considered a class effect of BCR pathway inhibitors, has been highlighted by several studies (Herman et al., 2014b; Farooqui et al., 2015; Thompson et al., 2015a) and was also confirmed in all three patient cohorts investigated in the present study. Despite this observation, we here report a different pattern of recirculation lymphocytosis upon ibrutinib treatment by comparing the median ALC kinetics of CLL cases expressing or not the VLA-4 α chain CD49d. In fact, the typical ibrutinib-induced early peak of lymphocytosis was found in CD49d^−^ CLL, but not in CD49d^+^ cases. Notably, such different behavior between CD49d^+^ and CD49d^−^ CLL was documented in cohorts with different mean ALCs before treatment and involved patients enrolled in different clinical trials, named patient programs (NPP), and from the real world, thus implying that the observed effects did not occur because of the selection of a particular group of patients. Interestingly, the ALC kinetics characterizing CD49d^+^ CLL were similar to those observed in previous studies of UM *IGHV* CLL and trisomy 12 CLL (Farooqui et al., 2015; Thompson et al., 2015a). Although data on CD49d expression were unavailable in these studies, it is known that almost all trisomy 12 CLL cases express CD49d and that CD49d^+^ CLL usually has UM *IGHV* (Gattei et al., 2008; Zucchetto et al., 2013; Dal Bo et al., 2016).

Undoubtedly, ibrutinib has exceptional activity in patients with RR CLL, as well as in untreated patients with genetic abnormalities that predict chemoresistance (i.e., *TP53* deletion or mutation), where a significant reduction in organomegaly and LN size is observed in most cases (Byrd et al., 2014). In the present study, analysis of nodal response in patients from a phase II clinical trial and an NPP confirmed the remarkable ibrutinib-mediated reduction of lymphadenopathies. Nevertheless, when comparing the extent of nodal response in CD49d^−^ and CD49d^+^ CLL, a trend of lower response was already observable after 2 mo of ibrutinib treatment in CD49d^+^ CLL, a difference that became significant after 6 and 12 mo. In contrast, no correlation between nodal response and other prognostic parameters, including *TP53* disruption and *IGHV* mutational status, was observed. An overall sustained nodal response also in cases without an apparent ALC change, as observed in CD49d^+^ CLL, can be explained by the gradual release of cells from nodal compartment because of the presence of a still-active VLA-4 integrin on the CLL cell surface. On the contrary, CD49d^−^ CLL cells, lacking one of the key molecules involved in cell adhesion, can be rapidly released into blood circulation, yielding the expected peak of redistribution lymphocytosis. Another possible mechanism explaining the lack of ALC rise in CD49d^+^ CLL relies upon the notion that CD49d^+^ CLL cells, being more addicted to microenvironmental-driven survival mechanisms, may more rapidly undergo cell death when released into the PB than CD49d^−^ CLL cells (Burger et al., 2017). A similar mechanism has been postulated to explain the prominent redistribution lymphocytosis observed in some instances in CLL patients with M *IGHV* (Woyach et al., 2014b).

Another observation of the present study is that the inverse relationship between ALC/SPD changes and CD49d expression values was mainly driven by negative/positive CD49d expression, thus emphasizing the relevance of the clinical cutoff established to identify CD49d^+^ CLL (Bulian et al., 2014). Similarly, evidence was provided that CD49d expression levels above the clinical cutoff, were necessary to effectively allow BCR-induced inside-out VLA-4 activation. In keeping with this line of reasoning, it has recently been shown that CLL cases expressing CD49d above the 30% of positive cells cutoff are characterized by a more frequent nodal presentation and subsequent development of lymphadenopathy (Strati et al., 2017).

Since the introduction of ibrutinib for CLL treatment, despite the durable clinical responses in the majority of patients, events of progression have started to be described, mainly because of the acquisition of *BTK* or *PLCγ2* mutations (Woyach et al., 2014a; Liu et al., 2015; Ahn et al., 2017). Here, we report that patient outcome after ibrutinib treatment can be affected by the presence of the VLA-4 integrin on the surface of CLL cells. Indeed, CD49d^+^ CLL were characterized by inferior PFS than CD49d^−^ CLL. Moreover, the concomitant presence of *TP53* disruption and high CD49d expression selected a patient subset at higher risk of progression, even if compared with cases with *TP53* disruption but lacking CD49d expression. In this regard, it is tempting to speculate that CLL cells with the concomitant presence of VLA-4, which is responsible for the retention of CLL cells in tissue sites, and *TP53* disruption, in turn responsible for genetic instability, may be particularly prone to develop ibrutinib resistance.

Collectively, the data presented in this study suggest that VLA-4–expressing CLL cells residing in the secondary lymphoid organs can receive BCR-mediated stimuli that are able to induce inside-out VLA-4 activation even in the presence of BTK blockade by ibrutinib. This activation leads to enhanced retention of VLA-4^+^ CLL cells in tissue sites with a parallel reduction of redistribution lymphocytosis and a relatively lower and/or slower nodal response eventually affecting patient outcome. It is tempting to speculate that the relative reduction of CD49d expression observed in PB CLL cells from ibrutinib-treated CD49d^+^ CLL cases, also documented in the context of the early released CLL cell subpopulations expressing the CXCR4^dim^/CD5^bright^ phenotype (Calissano et al., 2011), could be caused by the release from tissue sites of the cells with fewer surface CD49d molecules within the CLL clone, whereas CLL cells with the highest CD49d expression within the clone can be more likely retained in tissue sites. A formal proof for this hypothesis could be obtained by comparing CD49d expression and activation, before and after ibrutinib treatment, in the context of tissue sites.

From a practical standpoint, our data suggest that evaluation of CD49d expression in patients initiating ibrutinib therapy may identify those cases that would benefit from combination therapy approaches designed to completely block VLA-4 activation and VLA-4–mediated retention of leukemic cells in protective tissue compartments (de Rooij et al., 2015, 2016). Larger CLL cohorts or a more comprehensive characterization of CLL cases, which includes the novel genetic lesions or the detection of complex karyotype (Thompson et al., 2015b; Dal Bo et al., 2016), are needed to definitely validate CD49d as progression predictor in ibrutinib-treated CLL.

## Materials and methods

### CLL patients

This study included CLL patients treated with ibrutinib in the context of NPPs, clinical trials, or the real world, as follows: (1) 37 refractory/relapsed CLL patients enrolled in a multicenter Italian NPP and referred to the Clinical and Experimental Onco-Hematology Unit of the Aviano National Cancer Institute as reference laboratory (IT cohort); (2) 34 out of 86 CLL patients enrolled in the phase II monocentric trial NCT01500733 (NIH cohort). Patients from this cohort were chosen for the availability of CD49d expression data; no differences in terms of the patient’s characteristics (Table S2) were found between these patients and the remaining cases (Herman et al., 2014b); (3) 40 CLL patients, representing a consecutive cohort of patients enrolled by a single institution (Mayo Clinic, Rochester, MN) in the phase II multicenter trial NCT01744691 or in the phase III US-wide multicenter trials NCT01578707, NCT01886872, and NCT02048813 or treated with ibrutinib in the real-world (Mayo cohort); (4) 8 CD49d^+^ CLL patients from the phase II trial NCT02007044 provided by the MD Anderson Cancer Center (MDA cohort) solely used for in vitro experiments. Patients were treated with ibrutinib at a dose of 420 mg daily as a single agent, with the only exception of those enrolled in clinical trial NCT02048813 who also received rituximab starting from day 30.

In vitro studies were performed on PB mononuclear cells (PBMCs) and purified CLL cells from CD49d^+^ CLL of the IT and MDA cohorts collected pretreatment, and after days 7, 30, or 90 on ibrutinib, and from CD49d^+^ ibrutinib-naive CLL patients by taking advantage of the Aviano (Italy) and Salzburg (Austria) CLL biobanks.

Table S1 summarizes the main clinical and biological parameters, including CD49d expression, *IGHV* mutational status, and the main cytogenetic abnormalities of CLL cases entering this study. A CLL case was considered CD49d^+^ or *IGHV* mutated according to the 30% or 2% cutoffs, respectively, and cytogenetic categories were defined according to Döhner classification (Döhner et al., 2000; Gattei et al., 2008; Bulian et al., 2014).

All patient samples were collected after informed consent in accordance with the declaration of Helsinki and under approval of the specific local ethics committee (approval IRB-05-2010 and IRB-05-2015 of the Centro di Riferimento Oncologico, Aviano, and approvals 415-E/1287/4-2011 and 415-E/1287/13-2016 of the Salzburg ethics committee). All trials were registered at www.clinicaltrials.gov.

### PBMC isolation and CLL cell purification

PBMCs were isolated by density-gradient centrifugation (Ficoll Lymphocyte Separation Media, GE Healthcare), cryopreserved in 90% FBS (Sigma-Aldrich) plus 10% dimethyl sulfoxide (Sigma-Aldrich), and stored in liquid nitrogen until use. When reported, in vitro treatments were performed with 1 µM ibrutinib (Axon Medchem BV) for 1 h at 37°C and with 5 µg/ml F(ab)_2_ anti-IgM (catalog 109.006.129; Jackson ImmunoResearch), or 5 µg/ml F(ab)_2_ anti-IgD (catalog 2032–01; Southern Biotechnology) for the indicated time periods (Herman et al., 2015; Ten Hacken et al., 2016). For specific experiments, CLL cells were purified by negative selection using a mixture of anti-CD3, anti-CD16, and anti-CD14 mAbs (provided by F. Malavasi, University of Turin, Turin, Italy) and separation by immunomagnetic beads (Thermo Fisher Scientific) or left untouched when the CLL cells purity was >95% (Zucchetto et al., 2012). In all cases, B cell purity was >95%, as assessed by flow cytometry.

### Flow cytometry

For immunophenotypic evaluations, PBMCs from CLL samples were stained with anti-CD19 PE-Cy7 (catalog 560728; BD Biosciences) and anti-CD5 APC or CD5 FITC (catalog 555355 and 555352; BD Biosciences) mAbs, to identify CLL cells, with DAPI (Sigma-Aldrich) to exclude dead cells, and with anti-CD49d PE (catalog 555503; BD Biosciences), anti-CD29 PE (catalog 555443; BD Biosciences), anti-CXCR4 APC (catalog 555976; BD Biosciences), or anti-IgM FITC mAbs (catalog F0058; Dako). PBMCs from healthy donors were stained with anti-CD19 BV421 (catalog 562440; BD Biosciences), anti-CD49d PE, anti-CD29 PE, anti-IgG BB515 (catalog 564581; BD Biosciences), anti-IgM APC (catalog 551062; BD Biosciences). For phosphoprotein expression analysis, PBMCs (5 × 10^5^ per experimental condition), treated or not with 1 µM ibrutinib (1 h at 37°C) and stimulated for 10 min with either 5 µg/ml F(ab)_2_ anti-IgM or F(ab)_2_ isotypic control (catalog LS-C351727; LSBio), were stained with anti-CD3 BV605 (catalog 563219; BD Biosciences) and anti-CD19 BV421 mAbs and then fixed with Fix Buffer I (BD Biosciences) and permeabilized with 90% ice-cold methanol (VWR). Cells were labeled with one of the following mAbs: p-ERK (pT202/pY204) Alexa Fluor 488 (catalog 612592), p-BTK (pY223)/ITK (pY180)-PE (catalog 562753), p-PLCγ2 (pY759)-PE (catalog 558490), and p-AKT (pS473) Alexa Fluor 488 (catalog 560404) or Alexa Fluor 647 (catalog 560343; all from BD Biosciences). In all cases, setup experiments with appropriate isotype controls (catalogs 556650 and 557702) were performed to verify staining specificity. Cells were analyzed on a FACS Fortessa flow cytometer using FACS DIVA software (BD Biosciences) upon instrument calibration with CS&T beads (BD Biosciences). The specific fluorescence signals were recorded as absolute MFI values in log_10_ mode.

### Calcium assay

PBMCs (5 × 10^5^ per experimental condition) treated or not with 1 µM ibrutinib were incubated in RPMI medium (Sigma-Aldrich) and 1% FBS with 3 µM Fluo-4 AM (Thermo Fisher Scientific) for 30 min at 37°C. Cells were then washed, resuspended in RPMI with 1% FBS at 5 × 10^6^ cells/ml, and acquired at 37°C on FACSAria III (BD Biosciences). After a baseline acquisition for 1 min, cells were stimulated with 5 µg/ml F(ab)2 anti-IgM and recorded for another 3 min. To calculate the percentage of cells that exhibited increased fluorescence after addition of anti-IgM (percentage of responding cells), a background fluorescence threshold (T) at the fluorescence intensity of the 85th percentile of unstimulated cells was established for each sample, and the peak percentage of cells that exhibited an increase in fluorescence intensity above T after treatment with anti-IgM was then calculated (Mockridge et al., 2007). Data were analyzed by FlowJo software (Version 10; Tree Star).

### *TP53*, *BTK*, and *PLCγ2* mutational status

*TP53* mutational status was performed by a Sanger sequencing (exons 4–9; Rossi et al., 2013) or by NGS (exons 2–11) approaches. *BTK* C481S mutation and *PLCγ2* R665W, S707Y and L845F mutations were assessed by NGS. NGS analyses were run in a MiSeq sequencer (Illumina) using an amplicon-based strategy with at least 1,000× coverage and 1% of sensitivity. Primer sequences are available upon request. Data were analyzed with MiSeq reporter (Illumina) and IGV software (Thorvaldsdóttir et al., 2013) against human genome assembly hg19.

### VLA-4 activation assay

PBMCs (2 × 10^5^ per experimental condition) were incubated with a range of concentrations of the VLA-4–specific ligand, LDV-containing probe ((N'-2-methylphenyl ureido)-phenylacetyl-l-leucyl-l-aspartyl-l-valyl-l-prolyl-l-alanyl-l-alanyl-l-lysine, LDV; Commonwealth Biotechnologies) in the presence of an excess of anti-CD29 PE clone HUTS-21 mAb (catalog 556049; BD Biosciences), which specifically recognizes only the LDV-occupied VLA-4 conformation, for 30 min at 37°C, as described previously (Chigaev et al., 2009). A mixture of anti-CD3 FITC (catalog 555332; BD Biosciences) and anti-CD19 APC (catalog 555415; BD Biosciences) mAbs and DAPI were added during the last fifteen minutes incubation. Subsequently, the mean fluorescence intensity (MFI) of labeled HUTS-21 mAbs was measured, and VLA-4 RO was determined (ranging from 0.0 [no RO] to 1.0 [100% RO]). The criteria used to calculate the EC_50_ value and the resulting VLA-4-RO was the presence of increased HUTS-21 binding with increased LDV concentration. Under these conditions the EC_50_ of the HUTS-21–binding curve provides a measure of the VLA-4 ligand-binding affinity. RO was also used as an indicator of binding affinity (at 10 nM LDV), where higher RO indicates larger fraction of high-affinity receptors (Chigaev et al., 2009). When indicated, cells were treated with 5 µg/ml F(ab)_2_ anti-IgM during the above-reported 30-min incubation and/or ibrutinib (1 µM, 1 h pretreatment), as described previously (Herman et al., 2015).

### VLA-4 clustering assay

Falcon culture slides were coated with 7.5 µg/ml VCAM-1 (R&D Systems) overnight, washed, and blocked with 2% human serum albumin (Merck Millipore). Purified CLL cells were incubated for 1 h with or without 1 µM ibrutinib, added to the slides, and allowed to adhere for 30 min at 37°C in the presence or not of 5 µg/ml F(ab)_2_ anti-IgM before fixation with 4% paraformaldehyde. Slides were stained with primary anti-CD49d antibody (clone AHP1225; Bio-Rad) and Cy3-conjugated secondary anti–rabbit antibody. Images were taken with an Olympus IX81 microscope. For quantification, high-resolution images for cluster analysis were acquired on a Leica TCS SP5 II laser scanning microscope using a 63×/1.4-NA oil-immersion objective (Leica), and the number of clusters was analyzed using ImageJ software.

### Static adhesion assay

Negatively selected CLL cells treated or not with ibrutinib were labeled with the vital fluorochrome calcein AM (Thermo Fisher Scientific), seeded (2 × 10^5^ per experimental condition) in triplicate onto 96-well flat-bottom VCAM-1–coated plates (10 µg/ml; R&D Systems), and left to adhere for 30 min at 37°C. BSA-coated wells and polylysine-coated wells were used as negative and positive control of adhesion, respectively. The specificity of cell adhesion was checked by using anti-CD49d (clone P1H4) or anti-CD29 (clone 4B4) blocking mAbs (provided by A. Colombatti, CRO Aviano National Cancer Institute, Aviano, Italy), as previously reported (Zucchetto et al., 2016). Where indicated, 5 µg/ml F(ab)_2_ anti-IgM was added during this incubation period. Nonadhered cells were removed by washing with PBS and 1 mM Ca^2+^/Mg^2+^. The relative number of cells bound to the substrate was estimated by fluorescence detection in a computer-interfaced GeniusPlus microplate reader (Tecan). Results are reported as ratio of the mean of adherent cells on VCAM-1 over BSA ± SEM.

### Shear flow assay

Six-channel μ-slides (Ibidi) were coated with VCAM-1 (R&D Systems) at 4°C overnight, washed, and blocked with 2% human serum albumin. Purified CLL cells were incubated with or without 1 µM ibrutinib and 5 µg/ml F(ab)_2_ anti-IgM. The cells were perfused at 0.5 dyn/cm^2^ for 1 min at 37°C over the coated substrate. The perfusion period was recorded and digitalized. Analysis of the video-recorded segments was done using customized image analysis software (Wimasis). Arresting cells were defined as cells remaining stationary for at least 3 s on the substrate as previously described (Hartmann et al., 2009; Ganghammer et al., 2015).

### Clinical assessments

ALC data from the IT, NIH, and Mayo cohorts were collected before treatment and after 30, 60, 90, and 120 d on ibrutinib. The SPD of up to five LN regions (laterocervical, axillary, mediastinal, abdominal, and inguinal) was computed at 2 mo (NIH cohort), 6 mo (NIH cohort), and 12 mo (NIH and IT cohorts) on ibrutinib from computer tomography scan or physical examination. Patients undergoing progression and/or Richter syndrome transformation within 12 mo were excluded from SPD evaluation.

For clinical evaluations, PFS was defined as the time from the start of ibrutinib until progression or death (Cheson et al., 1999). Treatment response was evaluated according to 2008 revised International Workshop on CLL (IWCLL), incorporating the 2012 clarifications for patients treated with kinase inhibitors as previously reported (Hallek et al., 2008; Farooqui et al., 2015; Ahn et al., 2017). OS was defined as the time from the start of ibrutinib until last follow-up or death. Probability of PFS was estimated by the Kaplan–Meier method, and patients alive and progression-free were censored at the last follow-up. The log-rank test was used to compare PFS probabilities between subgroups. Median follow-up was computed using the OS database and by applying the inverted censoring method. Cox proportional hazards regression models were used to assess the independent effect of variables on PFS.

### Statistics

All statistical analyses were performed using MedCalc software (Mariakerke) and GraphPad Prism 5. All data were tested for normal distribution. Normally distributed data were compared using *t* tests (paired or unpaired), and nonparametric data were compared using the Mann–Whitney test (independent) or Wilcoxon high-rank test (paired analysis). Categorical data were compared with Fisher’s exact test. For all tests, significance levels were defined as *, P < 0.05; **, P < 0.01; ***, P < 0.001; and ****, P < 0.0001.

### Online supplemental material

Fig. S1 shows data on the effects of BCR triggering and of ibrutinib treatment in both normal B cells and CLL cells. Fig. S2 shows data of lymphocytosis in CD49d^+^ and CD49d^−^ CLL from the NCT02048813 clinical trial and the ibrutinib-induced nodal response in all CLL after 2, 6, and 12 mo of therapy and in CD49d^+^ and CD49d^−^ CLL after 2 mo and 6 mo of therapy. Fig. S3 shows correlation analyses between the levels of CD49d expression and ibrutinib-induced clinical effects. Table S1 lists the clinical and biological information of all CLL cases used in this study. Table S2 shows comparisons between the clinical and biological features of patients used in this study and those of the remaining patients from the phase II monocentric trial NCT01500733 (NIH cohort).

## Supplementary Material

Supplemental Materials (PDF)
