# Supplementary material for: Functional and clinical relevance of VLA-4 (CD49d/CD29) in ibrutinib-treated chronic lymphocytic leukemia
Source: J Exp Med. 2018 Feb 5;215(2):681–97. doi: 10.1084/jem.20171288 (PMC5789417; doi:10.1084/jem.20171288)
Supplement: Supplemental Materials (PDF) [file JEM_20171288_sm.pdf]

Journal of Experimental Medicine

SUPPLEMENTAL MATERIAL

Tissino et al., https://doi.org/10.1084/jem.20171288

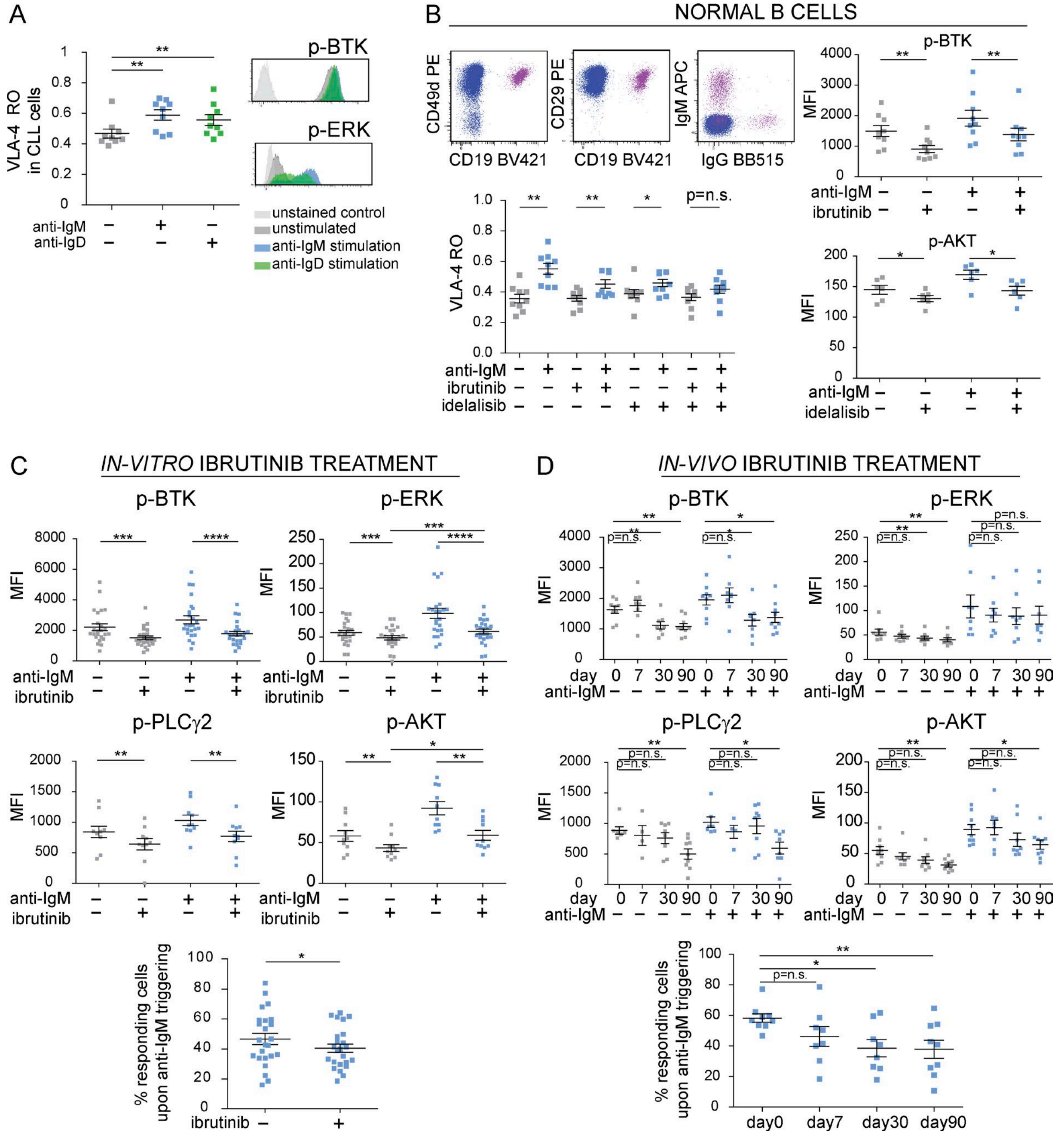

Figure S1. **Data on the effects of BCR triggering and of ibrutinib treatment in both normal B cells and CLL cells. (A)** VLA-4 RO in primary CLL cells from nine cases, stimulated with 5 µg/ml anti-IgM or anti-IgD. The histogram plot overlays on the right show phospho (p)-BTK and p-ERK expression in the unstimulated (gray), anti-IgM–stimulated (blue), and anti-IgD–stimulated (green) cells from one representative CLL case. light gray histogram represents the unstained control. **(B)** Top left: PBMCs from healthy donors (*n* = 9) were stained with anti-CD19 and anti-CD49d, anti-CD29, or a combination of anti-IgG and anti-IgM. Representative flow-cytometry dot plots for each staining from one representative donor are reported. Right: p-BTK and p-AKT MFI in unstimulated and anti-IgM–stimulated normal B cells from 9 healthy donors pretreated or not with either 1 µM ibrutinib (top) or 1 µM idelalisib (bottom). Bottom left: VLA-4 RO in unstimulated and anti-IgM–stimulated CLL cells untreated or treated with1 µM ibrutinib, 1 µM idelalisib, or a combination of both. **(C)** p-BTK (*n* = 24), p-ERK (*n* = 24), p-PLCγ2 (*n* = 10), and p-AKT (*n* = 10) MFI in unstimulated and anti-IgM–stimulated CLL cells treated or not with 1 µM ibrutinib. To facilitate comparisons, panels showing BCR response in the absence of ibrutinib treatment, as reported in Fig. 1 (B and C), are displayed. The bottom panel shows calcium response to anti-IgM stimulation in PBMCs from 24 CLL cases pretreated or not with 1 µM ibrutinib. **(D)** p-BTK, p-ERK, p-PLCγ2, and p-AKT MFI in unstimulated and anti-IgM–stimulated cells from CLL serial samples (*n* = 9) obtained before treatment (day 0) and at days 7, 30, and 90 on ibrutinib therapy. The bottom panel shows calcium response to anti-IgM stimulation. Data are presented as mean ± SEM. Individual symbols represent individual cases. *, P < 0.05; **, P < 0.01; ***, P < 0.001; ****, P < 0.0001; n.s., not significant (Wilcoxon test).

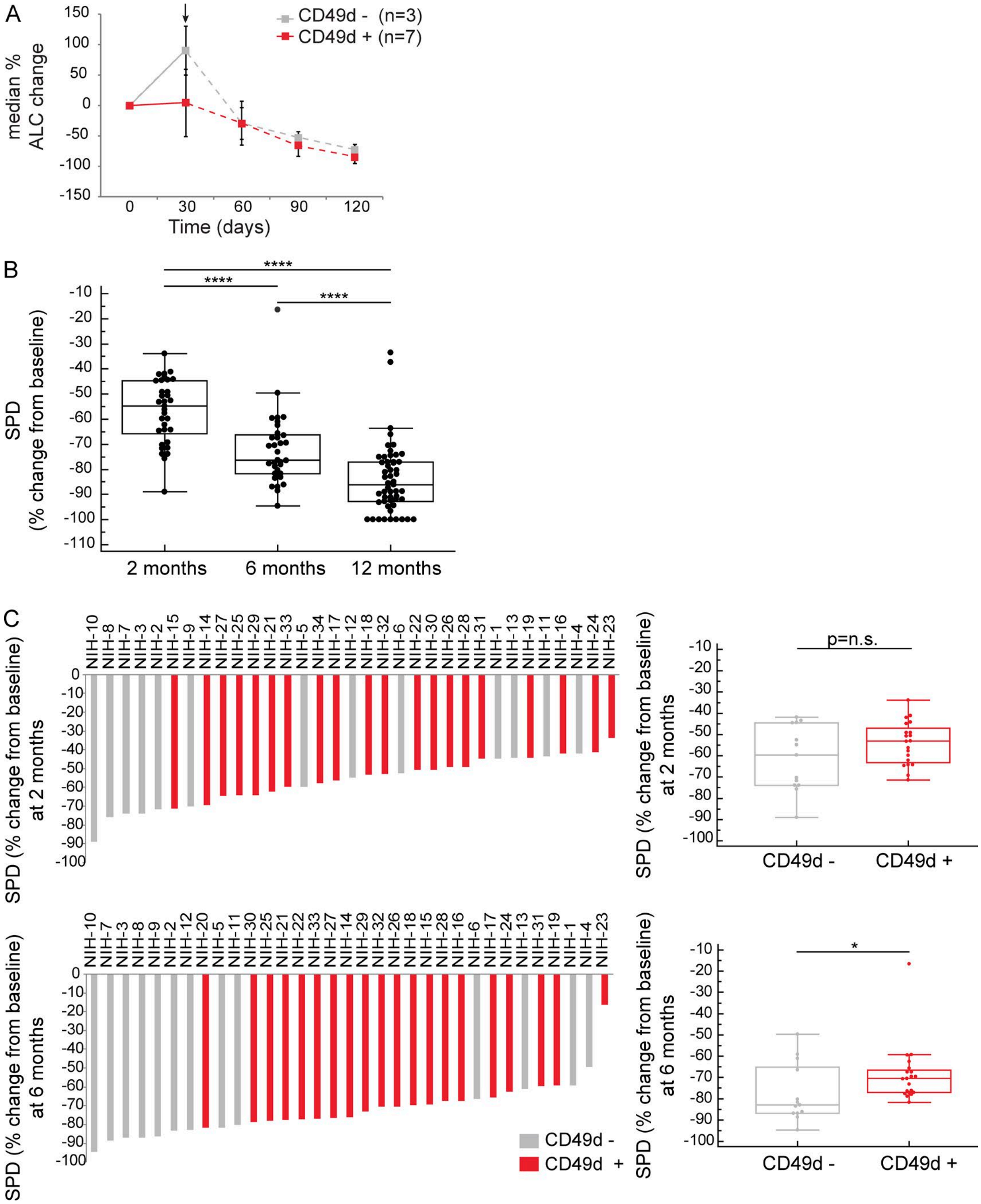

Figure S2. **Data of lymphocytosis in CD49d⁺ and CD49d⁻ CLL from the NCT02048813 clinical trial and the ibrutinib-induced nodal response in all CLL after 2, 6, and 12 mo of therapy and in CD49d⁺ and CD49d⁻ CLL after 2 mo and 6 mo of therapy. (A)** Redistribution lymphocytosis in CD49d⁺ and CD49d- CLL from the NCT02048813 clinical trial (n = 10). Absolute lymphocyte counts (ALC) were collected pretreatment (day 0) and at different treatment time points (day 30, 60, 90, and 120) in CLL cases from the NCT02048813 clinical trial. The line graphs show the median percent ALC change from baseline in CLL cases split according to CD49d expression (CD49d⁺, red line; CD49d⁻, gray line); the black vertical lines indicate SEM. The full lines indicate the ibrutinib treatment as single agent, whereas the dashed lines correspond to the addition of rituximab to the treatment at day 30 (arrow); the number of patients included in each group are reported in parentheses. **(B)** LN dimension evaluation was performed pretreatment and after 2, 6, and 12 mo of ibrutinib therapy, and the SPD of up to 5 LN regions was calculated. The box and whisker plot shows the median percent SPD change at 2, 6, and 12 mo of ibrutinib therapy in all CLL cases (n = 33, n = 33, and n = 53, respectively). **(C)** The waterfall plots show the percent SPD change from baseline at 2 mo (upper plot) and at 6 mo (lower plot) in CD49d⁺ (red bars) or CD49d⁻ (gray bars) CLL cases. The box and whisker plots on the right show the median percent SPD change at 2 and 6 mo of ibrutinib therapy in CD49d⁻ (n = 13, gray box) and CD49d⁺ (n = 20, red box) CLL cases. *, $P < 0.05$; ****, $P < 0.0001$; n.s., not significant (Mann–Whitney test).

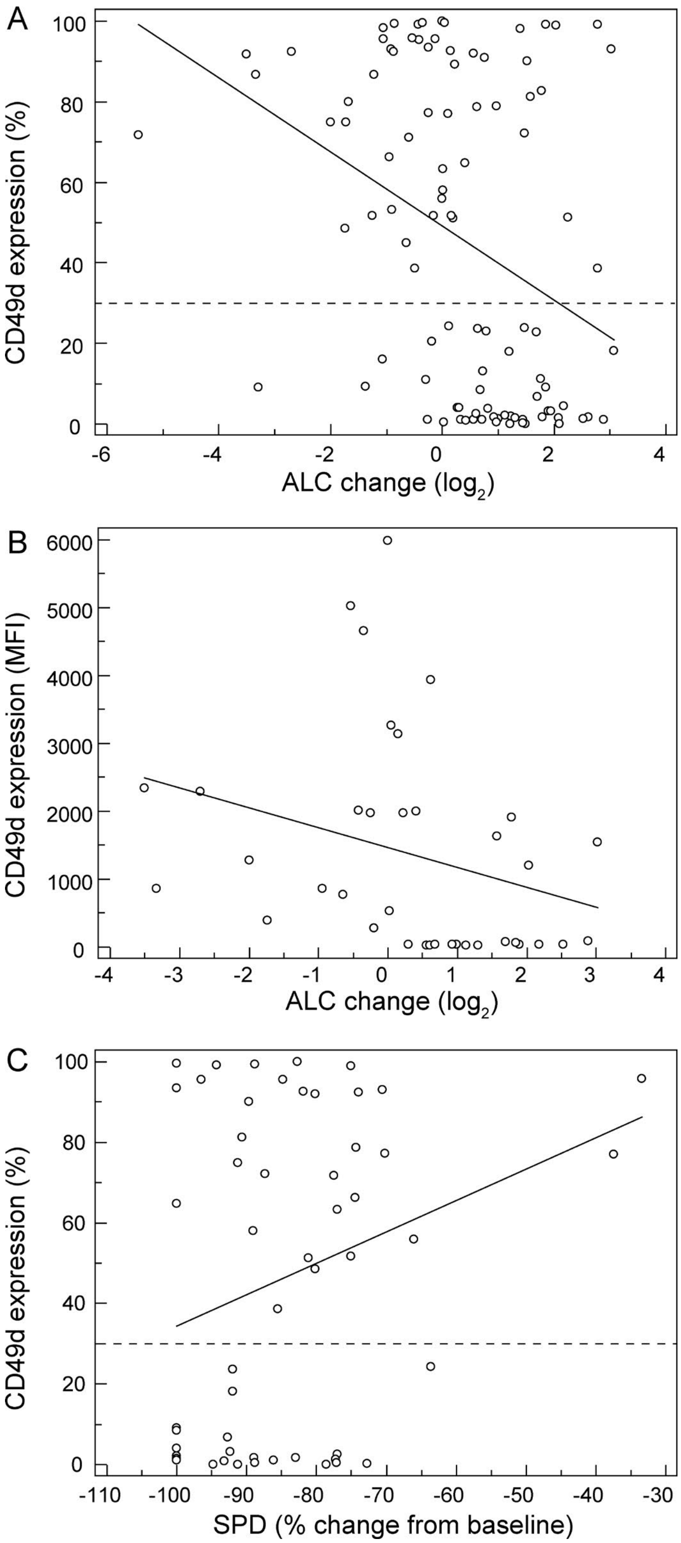

Figure S3. **Correlation analyses between the levels of CD49d expression and ibrutinib-induced clinical effects. (A–C)** Scatterplots display the correlations between the percentage of CD49d expression and ALC change (r = −0.36, P = 0.0003; *n* = 100, IT, NIH, and Mayo cohorts; A), CD49d MFI and ALC change (r=-0.29, P = 0.07; *n* = 37, IT cohort; B), and percentage of CD49d expression and percent SPD change from the baseline (r = 0.27, P = 0.04; *n* = 53, IT and NIH cohorts; C). The ALC change was computed by applying the formula log2(ALCt30/ALCt0). In each plot, the regression line is reported; the dashed lines indicate the 30% cutoff of CD49d expression.

Table S1. **Patient characteristics**

Case[a]	Age	Sex	% CD49d[b]	Rai stage	*IGHV*	*TP53*	FISH[c]	β2M (mg/l)	WBC ($\times10^9$/l)	TN	LN evaluation at 12 mo	ALC values available	Ibrutinib treatment	PFS data available[d]
IT-1[e]	54	f	4	I	M	mut	del17p	1.9	100.0	No	Yes	Yes	NPP	Yes
IT-2[e]	53	f	21	III	UM	WT	del11q	na	96.0	No	No	Yes	NPP	Yes
IT-3	42	m	2	I	UM	mut	del13q	1.9	23.2	No	Yes	Yes	NPP	Yes
IT-4	51	m	2	I	UM	WT	del11q	2.6	98.0	No	Yes	Yes	NPP	Yes
IT-5[e]	59	f	9	I	UM	WT	del11q	1.7	45.0	No	Yes	Yes	NPP	Yes
IT-6	59	f	5	II	M	WT	del13q	2.3	7.0	No	No	Yes	NPP	Yes
IT-7	38	f	2	I	UM	WT	del13q	1.7	26.0	No	Yes	Yes	NPP	Yes
IT-8	65	m	1	II	M	mut	del13q	2.3	20.0	No	Yes	Yes	NPP	Yes
IT-9	59	m	3	I	UM	WT	del13q	1.4	15.1	No	Yes	Yes	NPP	Yes
IT-10	51	m	7	I	UM	WT	del11q	2.1	9.5	No	Yes	Yes	NPP	Yes
IT-11	47	m	3	II	UM	WT	del17p	2.2	5.4	No	No	Yes	NPP	Yes
IT-12	56	m	9	I	UM	WT	del11q	2.3	3.5	No	Yes	Yes	NPP	Yes
IT-13	66	m	1	II	UM	WT	del11q	2.1	4.7	No	Yes	Yes	NPP	Yes
IT-14	51	f	0	IV	UM	WT	del11q	na	90.9	No	Yes	Yes	NPP	Yes
IT-15	na	m	0	na	UM	mut	na	4.3	47.5	No	No	Yes	NPP	Yes
IT-16[e]	na	m	25	na	UM	na	del11q	na	na	N/A	No	No	NT	N/A
IT-17[e]	na	f	14	na	M	WT	norm	na	na	N/A	No	No	NT	N/A
IT-18[e]	na	m	4	na	UM	WT	norm	na	na	N/A	No	No	NT	N/A
IT-19[e]	62	f	4	I	UM	na	del17p	na	na	N/A	No	No	NT	N/A
IT-20[e]	na	m	18	na	M	na	del17p	na	na	N/A	No	No	NT	N/A
IT-21[e]	na	m	13	na	UM	WT	norm	na	na	N/A	No	No	NT	N/A
IT-22[e,f]	64	f	63	0	UM	mut	del11q	1.5	127.3	No	Yes	Yes	NPP	Yes
IT-23[e,f]	58	m	93	II	UM	WT	del11q	7.3	10.7	No	No	Yes	NPP	Yes
IT-24[e,f]	65	m	83	I	UM	WT	del13q	na	na	No	No	Yes	NPP	Yes
IT-25	67	m	93	II	UM	mut	del17p	5.4	50.0	No	Yes	Yes	NPP	Yes
IT-26	56	f	93	II	M	WT	del11q	3.3	70.2	No	Yes	Yes	NPP	Yes
IT-27[e,f]	62	f	100	I	M	WT	tri12	2.2	63.5	No	Yes	Yes	NPP	Yes
IT-28	69	f	100	II	UM	mut	del11q	3.1	9.6	No	No	Yes	NPP	Yes
IT-29[e,f]	49	m	66	II	UM	WT	tri12	3.4	32.7	No	Yes	Yes	NPP	Yes
IT-30	65	f	87	I	UM	na	tri12	3.2	36.3	No	No	Yes	NPP	Yes
IT-31	62	f	100	II	UM	WT	norm	na	na	No	Yes	Yes	NPP	Yes
IT-32[e]	59	m	96	II	UM	mut	del17p	4.0	6.8	No	Yes	Yes	NPP	Yes
IT-33	na	f	45	na	UM	mut	del17p	3.9	127.5	No	No	Yes	NPP	Yes
IT-34	58	f	65	II	UM	mut	del17p	3.4	4.7	No	Yes	Yes	NPP	Yes
IT-35[e,f]	56	m	99	II	UM	mut	del17p	3.4	10.5	No	Yes	Yes	NPP	Yes
IT-36	48	f	49	I	UM	na	del17p	na	228.0	No	Yes	Yes	NPP	Yes
IT-37[e,f]	na	m	99	na	UM	mut	del17p	na	58.0	No	No	Yes	NPP	Yes
IT-38[e]	74	m	100	0–I	M	WT	norm	na	na	N/A	No	No	NT	N/A
IT-39[e]	na	m	100	na	M	na	del13q	na	na	N/A	No	No	NT	N/A
IT-40[e]	51	f	100	II	UM	WT	norm	na	na	N/A	No	No	NT	N/A
IT-41[e]	84	m	98	0	UM	na	tri12	na	na	N/A	No	No	NT	N/A
IT-42[e]	na	m	97	na	UM	na	del11q	na	na	N/A	No	No	NT	N/A
IT-43[e]	76	m	97	II	M	WT	del13q	na	na	N/A	No	No	NT	N/A
IT-44[e]	65	m	97	I	UM	mut	del17p	na	na	N/A	No	No	NT	N/A
IT-45[e]	70	m	93	I	UM	na	del13q	na	na	N/A	No	No	NT	N/A
IT-46[e]	na	f	93	na	UM	WT	na	na	na	N/A	No	No	NT	N/A
IT-47[e]	64	m	87	0	UM	na	na	na	na	N/A	No	No	NT	N/A
IT-48[e]	na	f	82	na	M	na	del13q	na	na	N/A	No	No	NT	N/A
IT-49[e]	60	f	79	I	UM	WT	del13q	na	na	N/A	No	No	NT	N/A
IT-50[e]	61	m	75	II	M	WT	del13q	na	na	N/A	No	No	NT	N/A
IT-51[e]	51	m	100	II	UM	na	del11q	na	na	N/A	No	No	NT	N/A
IT-52	49	f	79	IV	UM	na	del17p	na	19.7	No	Yes	Yes	NPP	Yes
IT-53	57	m	92	II	UM	WT	tri12	na	168.4	No	No	Yes	NPP	Yes
IT-54	na	m	92	na	UM	WT	del11q	na	18.5	No	No	Yes	NPP	Yes
IT-55	64	m	89	na	UM	mut	norm	na	44.0	No	No	Yes	NPP	Yes
IT-56	na	m	81	na	UM	na	del17p	na	4.7	No	Yes	Yes	NPP	Yes
IT-57	63	f	75	I	UM	na	del11q	na	4.0	No	Yes	Yes	NPP	Yes
IT-58[e]	61	m	100	na	M	na	norm	na	na	N/A	No	No	NT	N/A
IT-59[e]	87	f	79	0–I	UM	na	del17p	na	na	N/A	No	No	NT	N/A
IT-60[e]	72	f	95	IV	UM	na	del11q	na	na	N/A	No	No	NT	N/A
IT-61[e]	74	m	68	II	M	na	del13q	na	na	N/A	No	No	NT	N/A

Table S1. **Patient characteristics** *(Continued)*

Case[a]	Age	Sex	% CD49d[b]	Rai stage	*IGHV*	*TP53*	FISH[c]	β2M (mg/l)	WBC ($\times 10^9$/l)	TN	LN evaluation at 12 mo	ALC values available	Ibrutinib treatment	PFS data available[d]
IT-62[e]	84	m	100	0	UM	na	tri12	na	na	N/A	No	No	NT	N/A
IT-63[e]	na	m	35	na	M	WT	norm	na	na	N/A	No	No	NT	N/A
IT-64[e]	na	m	40	na	M	na	del11q	na	na	N/A	No	No	NT	N/A
IT-65[e]	na	m	40	na	UM	mut	del17p	na	na	N/A	No	No	NT	N/A
IT-66[e]	na	m	50	na	na	WT	tri12	na	na	N/A	No	No	NT	N/A
MDA-1[e]	na	na	63	na	na	na	na	na	na	No	No	No	NCT02007044	No
MDA-2[e]	na	na	97	na	UM	na	del13q	na	na	No	No	No	NCT02007044	No
MDA-3[e]	na	na	80	na	UM	na	del17p	na	na	No	No	No	NCT02007044	No
MDA-4[e]	na	na	75	na	UM	na	del11q	na	na	No	No	No	NCT02007044	No
MDA-5[e]	na	na	85	na	na	na	del17p	na	na	No	No	No	NCT02007044	No
MDA-6[e]	na	na	65	na	UM	na	norm	na	na	No	No	No	NCT02007044	No
MDA-7[e]	na	na	59	na	UM	na	del11q	na	na	No	No	No	NCT02007044	No
MDA-8[e]	na	na	91	na	UM	na	del17p	na	na	No	No	No	NCT02007044	No
MDA-9[e]	na	na	100	na	UM	na	del17p	na	na	No	No	No	NCT02007044	No
NIH-1	74	m	0	IV	M	WT	del13q	4.4	127.5	Yes	Yes[g,h]	Yes	NCT01500733	Yes
NIH-2	59	m	0	I	UM	mut	del17p	5.2	65.2	No	Yes[g,h]	Yes	NCT01500733	Yes
NIH-3	66	m	0	IV	M	mut	del17p	3.4	43.6	Yes	Yes[g,h]	Yes	NCT01500733	Yes
NIH-4	82	m	0	IV	M	mut	del17p	4.9	79.9	Yes	Yes[g,h]	Yes	NCT01500733	Yes
NIH-5	33	m	0	II	UM	mut	del17p	1.7	52.9	Yes	Yes[g,h]	Yes	NCT01500733	Yes
NIH-6	60	f	1	I	M	mut	del17p	1.7	63.0	Yes	Yes[g,h]	Yes	NCT01500733	Yes
NIH-7	69	m	1	I	M	WT	del13q	4.4	16.7	Yes	Yes[g,h]	Yes	NCT01500733	Yes
NIH-8	67	m	2	III	UM	WT	del11q	7.8	96.1	No	Yes[g,h]	Yes	NCT01500733	Yes
NIH-9	66	f	3	I	M	mut	del17p	3.8	35.8	No	Yes[g,h]	Yes	NCT01500733	Yes
NIH-10	78	m	9	I	UM	WT	del13q	5.1	74.3	No	No[g,h]	Yes	NCT01500733	Yes
NIH-11	56	m	18	IV	M	mut	del17p	7.1	3.5	No	Yes[g,h]	Yes	NCT01500733	Yes
NIH-12	58	f	24	III	UM	mut	del17p	4.0	68.4	Yes	Yes[g,h]	Yes	NCT01500733	Yes
NIH-13	72	m	24	0	M	WT	del13q	1.9	87.4	Yes	Yes[g,h]	Yes	NCT01500733	Yes
NIH-14	65	m	39	IV	M	WT	del11q	4.1	20.9	No	Yes[g,h]	Yes	NCT01500733	Yes
NIH-15	67	f	51	I	UM	mut	del17p	10.3	15.0	No	Yes[g,h]	Yes	NCT01500733	Yes
NIH-16	69	m	52	IV	M	WT	tri12	3.3	187.9	Yes	No[g,h]	Yes	NCT01500733	Yes
NIH-17	62	m	52	IV	M	mut	del17p	2.5	93.7	No	Yes[g,h]	Yes	NCT01500733	Yes
NIH-18	66	m	52	IV	UM	mut	del17p	8.7	146.4	No	No[g,h]	Yes	NCT01500733	Yes
NIH-19	62	f	56	III	UM	mut	del17p	7.6	101.1	No	Yes[g,h]	Yes	NCT01500733	Yes
NIH-20	66	m	58	III	UM	mut	del17p	6.5	35.4	No	Yes[g]	Yes	NCT01500733	Yes
NIH-21	73	m	72	IV	UM	mut	del17p	4.2	88.2	No	Yes[g,h]	Yes	NCT01500733	Yes
NIH-22	66	f	72	I	M	WT	del13q	2.4	29.1	No	Yes[g,h]	Yes	NCT01500733	Yes
NIH-23	77	m	77	IV	M	mut	del17p	4.6	33.3	Yes	Yes[g,h]	Yes	NCT01500733	Yes
NIH-24	77	m	77	IV	UM	WT	tri12	5.0	165.4	No	Yes[g,h]	Yes	NCT01500733	Yes
NIH-25	69	f	90	III	UM	WT	del11q	6.9	75.6	No	Yes[g,h]	Yes	NCT01500733	Yes
NIH-26	69	m	92	IV	M	WT	del11q	3.1	89.9	Yes	Yes[g,h]	Yes	NCT01500733	Yes
NIH-27	60	f	92	IV	UM	mut	del17p	8.6	111.1	No	Yes[g,h]	Yes	NCT01500733	Yes
NIH-28	79	m	93	IV	UM	mut	del17p	9.0	216.4	No	Yes[g,h]	Yes	NCT01500733	Yes
NIH-29	73	f	96	III	UM	WT	del11q	7.9	184.8	No	Yes[g,h]	Yes	NCT01500733	Yes
NIH-30	59	m	96	IV	M	mut	del17p	3.9	401.9	No	Yes[g,h]	Yes	NCT01500733	Yes
NIH-31	85	f	98	IV	UM	WT	tri12	5.8	131.1	No	Yes[g,h]	Yes	NCT01500733	Yes
NIH-32	70	f	99	IV	UM	WT	del13q	11.3	77.7	No	Yes[g,h]	Yes	NCT01500733	Yes
NIH-33	65	m	99	IV	UM	WT	tri12	4.2	149.4	No	Yes[g,h]	Yes	NCT01500733	Yes
NIH-34	68	f	39	IV	UM	WT	del13q	3.3	281.1	Yes	No[h]	Yes	NCT01500733	Yes
MAYO-1	66	f	13	IV	UM	na	norm	na	101.2	No	No	Yes	NCT01578707	Yes
MAYO-2	67	m	2	II	M	na	na	na	31.6	No	No	Yes	NCT01744691	Yes
MAYO-3	72	m	9	IV	UM	na	del11q	10.8	140.6	Yes	No	Yes	NCT01886872	Yes
MAYO-4	70	m	23	IV	M	na	del13q	7.3	77.4	Yes	No	Yes	NCT01886872	Yes
MAYO-5	75	m	1	IV	M	na	del13q	7.6	202.3	Yes	No	Yes	NCT01886872	Yes
MAYO-6	68	f	1	III	UM	na	del13q	8.9	264.6	Yes	No	Yes	NCT01886872	Yes
MAYO-7	64	f	4	II	UM	mut	na	na	352.7	No	No	Yes	RW	Yes
MAYO-8	66	m	23	IV	UM	na	del13q	na	26.3	No	No	Yes	RW	Yes
MAYO-9	77	m	1	IV	UM	na	del11q	na	27.6	No	No	Yes	RW	Yes
MAYO-10	53	m	18	II	M	na	del13q	na	76.5	No	No	Yes	RW	Yes
MAYO-11	82	f	11	III	M	na	del13q	na	18.7	No	No	Yes	RW	Yes
MAYO-12	67	f	2	IV	M	na	tri12	na	68.7	No	No	Yes	RW	Yes
MAYO-13	81	f	24	IV	M	na	na	na	73.0	No	No	Yes	RW	Yes

Table S1. **Patient characteristics** *(Continued)*

Case[a]	Age	Sex	% CD49d[b]	Rai stage	*IGHV*	*TP53*	FISH[c]	β2M (mg/l)	WBC (×10^9/l)	TN	LN evaluation at 12 mo	ALC values available	Ibrutinib treatment	PFS data available[d]
MAYO-14	57	m	2	IV	UM	mut	na	na	2.5	No	No	Yes	RW	Yes
MAYO-15	63	f	4	I	UM	na	del13q	na	123.9	No	No	Yes	RW	Yes
MAYO-16	63	f	1	II	M	WT	na	na	83.4	No	No	Yes	RW	Yes
MAYO-17	74	m	11	III	M	mut	del13q	na	76.8	Yes	No	Yes	RW	Yes
MAYO-18	80	f	16	IV	M	na	del11q	na	24.8	No	No	Yes	RW	Yes
MAYO-19	70	m	95	IV	UM	WT	norm	na	141.3	No	No	Yes	NCT01578707	Yes
MAYO-20	70	m	51	IV	UM	na	na	na	67.4	No	No	Yes	NCT01744691	Yes
MAYO-21	71	m	80	II	UM	na	del17p	17.2	11.4	Yes	No	Yes	NCT01886872	Yes
MAYO-22	73	m	87	IV	UM	na	tri12	5.9	19.8	Yes	No	Yes	NCT01886872	Yes
MAYO-23	79	m	53	IV	M	na	del13q	5.8	85.8	Yes	No	Yes	NCT01886872	Yes
MAYO-24	74	m	98	I	UM	mut	norm	7.4	19.0	Yes	No	Yes	NCT01886872	Yes
MAYO-25	72	m	91	I	M	na	del13q	na	173.8	Yes	No	Yes	RW	Yes
MAYO-26	53	f	75	III	UM	WT	na	na	36.8	No	No	Yes	RW	Yes
MAYO-27	62	m	79	0	UM	na	del13q	na	1.6	No	No	Yes	RW	Yes
MAYO-28	73	f	98	IV	M	na	norm	na	14.5	No	No	Yes	RW	Yes
MAYO-29	74	f	71	III	UM	mut	na	4.6	0.8	No	No	Yes	RW	Yes
MAYO-30	81	m	99	IV	M	na	tri12	na	20.5	No	No	Yes	RW	Yes
MAYO-31	35	f	3	I	UM	na	norm	3.0	na	Yes	No	Yes	NCT02048813	No
MAYO-32	59	m	1	IV	M	na	del13q	3.7	na	Yes	No	Yes	NCT02048813	No
MAYO-33	55	f	3	III	UM	na	del11q	4.7	na	Yes	No	Yes	NCT02048813	No
MAYO-34	65	m	100	IV	M	WT	tri12	5.2	na	Yes	No	Yes	NCT02048813	No
MAYO-35	59	f	34	IV	M	na	del13q	3.8	na	Yes	No	Yes	NCT02048813	No
MAYO-36	50	m	98	IV	UM	na	del13q	3.0	na	Yes	No	Yes	NCT02048813	No
MAYO-37	49	f	66	III	UM	na	norm	3.7	na	Yes	No	Yes	NCT02048813	No
MAYO-38	70	m	86	II	UM	na	tri12	10.5	na	Yes	No	Yes	NCT02048813	No
MAYO-39	60	m	100	I	UM	WT	del11q	5.8	na	Yes	No	Yes	NCT02048813	No
MAYO-40	56	m	99	III	UM	na	tri12	14.4	na	Yes	No	Yes	NCT02048813	No

β2M, β2 microglobulin; del13q, 13q14.3 deletion; del17p, 17p13.1 deletion; del11q, 11q22-q23 deletion; f, female; FISH, fluorescence in situ hybridization; m, male; M, mutated *IGHV*; mut, mutated *TP53*; tri12, trisomy 12; norm, normal, none of the above reported cytogenetic aberrations; N/A, not applicable; na, not available; NPP, named patient program; NT, not treated; RW, ibrutinib treatment in the real world; UM, unmutated *IGHV*; WBC, white blood cell; WT, WT *TP53*.

[a]IT denotes patients from the Italian cohort; MDA, patients from MD Anderson Cancer Center; NIH, patients from the National Heart, Lung and Blood Institute; MAYO, patients from the Mayo Clinic College of Medicine.

[b]Values indicate the percentage of CD49d-positive cells as computed in the $CD5^+CD19^+$ cell population; cases with CD49d expression values ≥30% were scored as $CD49d^+$; cases with CD49d expression values <30% were scored as $CD49d^-$.

[c]Chromosome aberrations were classified according to Döhner et al (2000).

[d]PFS was defined as the time from the start of ibrutinib until progression/death or last follow-up.

[e]Cases used for in vitro experiments.

[f]Cases evaluated for *BTK* and *PLCγ2* mutations.

[g]CT scan available at 6 mo.

[h]CT scan available at 2 mo.

Table S2. **Trial NCT01500733 patient characteristics**

Parameter	Patients used in our study (NIH cohort), *n* = 34	Remaining patients, *n* = 52	P
Median age, yr	67	65	NS[a]
Median ALC before treatment, mo	89	89.9	NS[a]
Male, n (%)	22 (64.7)	29 (55.8)	NS[b]
Rai stage III–IV, *n* (%)	25 (73.5)	35 (67.3)	NS[b]
UM *IGHV*, *n* (%)	19 (55.9)	38 (73.1)	NS[b]
TP53 disrupted, *n* (%)	18 (52.9)	35 (67.3)	NS[b]

[a]Unpaired Student's *t* test.

[b]Fisher's exact test.
